# Molecular basis of cholesterol efflux via ABCG subfamily transporters

**DOI:** 10.1073/pnas.2110483118

**Published:** 2021-08-17

**Authors:** Yingyuan Sun, Jin Wang, Tao Long, Xiaofeng Qi, Linda Donnelly, Nadia Elghobashi-Meinhardt, Leticia Esparza, Jonathan C. Cohen, Xiao-Song Xie, Helen H. Hobbs, Xiaochun Li

**Affiliations:** ^a^Department of Molecular Genetics, University of Texas Southwestern Medical Center, Dallas, TX 75390;; ^b^Eugene McDermott Center for Human Growth and Development, University of Texas Southwestern Medical Center, Dallas, TX 75390;; ^c^Institute of Chemistry, Technical University Berlin, 10623 Berlin, Germany;; ^d^Center for Human Nutrition, University of Texas Southwestern Medical Center, Dallas, TX 75390;; ^e^Howard Hughes Medical Institute, University of Texas Southwestern Medical Center, Dallas, TX 75390;; ^f^Department of Biophysics, University of Texas Southwestern Medical Center, Dallas, TX 75390

**Keywords:** ABCG5, ABCG8, ABCG1, plant sterol, sitosterolemia

## Abstract

The ABCG1 homodimer (G1) and ABCG5–ABCG8 heterodimer (G5G8), two members of the adenosine triphosphate (ATP)–binding cassette (ABC) transporter G family, are required for maintenance of cellular cholesterol levels. G5G8 mediates secretion of neutral sterols into bile and the gut lumen, whereas G1 transports cholesterol from macrophages to high-density lipoproteins (HDLs). The mechanisms used by G5G8 and G1 to recognize and export sterols remain unclear. Here, we report cryoelectron microscopy (cryo-EM) structures of human G5G8 in sterol-bound and human G1 in cholesterol- and ATP-bound states. Both transporters have a sterol-binding site that is accessible from the cytosolic leaflet. A second site is present midway through the transmembrane domains of G5G8. The Walker A motif of G8 adopts a unique conformation that accounts for the marked asymmetry in ATPase activities between the two nucleotide-binding sites of G5G8. These structures, along with functional validation studies, provide a mechanistic framework for understanding cholesterol efflux via ABC transporters.

## Significance

Cholesterol is an essential component of animal cell membranes whose level in cells is maintained within a narrow range. Cholesterol is actively excreted from cells by two ATP-binding cassette (ABC) transporters, ABCG5–ABCG8 (G5G8) in the liver and gut and ABCG1 (G1) in macrophages. The mechanism(s) by which these proteins translocate rigid, planar sterol molecules across the membrane bilayer remain unknown. Here, we report the structure of human G1 and G5G8 in their unbound and cholesterol-bound states. We also determined the structure of G1 bound to ATP. These structures, together with functional studies in model organisms and biochemical studies, identify the binding site for cholesterol and provide the basis for a model of cholesterol transport by ABC transporters.

Cholesterol is an essential constituent of cell membranes and can be synthesized from acetate by all nucleated cells in vertebrates. To maintain cholesterol homeostasis, the amount of cholesterol acquired by cells through de novo synthesis or lipoprotein uptake must be tightly coupled to the amount that is lost through degradation or excretion (1). The pathways by which cholesterol is synthesized, and the mechanisms by which these pathways are regulated, have been elucidated in considerable detail (1). Less is known about the molecular basis of cholesterol excretion. The observation that cholesterol flips rapidly between the inner and outer leaflets of phospholipid bilayers suggested that cholesterol efflux from cells does not require a protein mediator (2), but studies in humans with rare disorders of cholesterol metabolism provide evidence for active excretion of cholesterol from cells. Loss-of-function mutations in ABCA1 (adenosine triphosphate [ATP]–binding cassette transporter A1) cause Tangier disease, a disorder characterized by very low concentrations of circulating low-density lipoproteins and high-density lipoproteins (HDLs) and accumulation of cholesterol in macrophages (3–5). Mutations in ABCG5 (G5) or ABCG8 (G8) result in an autosomal recessive disorder, sitosterolemia, which is characterized by accumulation of both plant- (e.g., sitosterol and campesterol) and animal-derived sterols (e.g., cholesterol) as well as premature atherosclerosis (6, 7). Subsequently, genetic manipulation studies in mice indicated that ABCG1 (G1) also transports cholesterol (8).

**Fig. 1.**
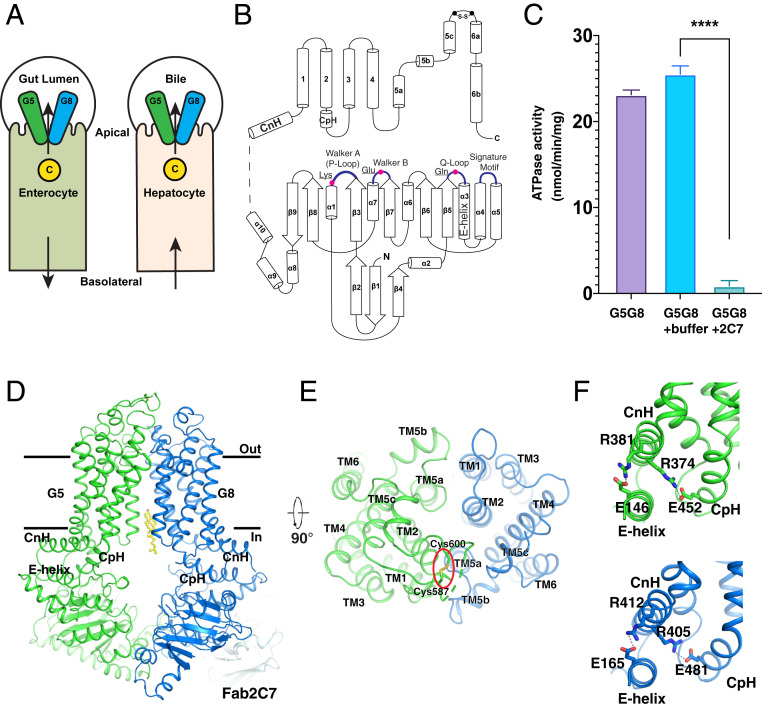
Overview of human G5G8 and the effect of Fab2C7 binding on ATPase activity. (*A*) Localization of G5G8 on apical (biliary) membrane of hepatocytes and enterocytes. The cellular cholesterol (C) is indicated. (*B*) Topology diagram of an ABCG transporter. The linker between the NBD and TMHs, which is not resolved in the structure, is shown as a dashed line. The secondary and key structural elements and residues are indicated. (*C*) The ATPase activity of purified G5G8 is blocked by Fab2C7. ****P < 0.0001, *t* test using GraphPad Prism 7; data are mean ± SD (*n* = 4 to 6 per group). (*D*) Overall structure of the G5G8–Fab2C7 complex viewed from the side of the membrane. The CpH, CnH, and E helix in G5 and G8 are indicated. The cholesterol in sterol-binding site 1 is shown as yellow sticks. (*E*) The extracellular view of the TMHs. The disulfide bond is indicated by a red oval. (*F*) The interaction details of the buried salt bridges in the three-helix bundles.

Members of the G subfamily of ABC transporters are encoded as hemitransporters and dimerize in order to function (9, 10). G5 and G8 heterodimerize before exiting the endoplasmic reticulum and trafficking to the apical membrane, where they mediate excretion of cholesterol into bile and the intestinal lumen (Fig. 1*A*) (6, 11, 12). G1 is abundantly expressed in macrophages and exports cellular cholesterol to extracellular acceptors, especially HDL (8, 13–15). G1 has been reported to facilitate reverse cholesterol transport (16, 17), the pathway by which cholesterol made in peripheral tissues is transported back to the liver or to the gut for excretion (18).

A schematic of a canonical ABCG half-transporter is shown in Fig. 1*B*. Each half-transporter contains a Walker A (P loop), Q loop, Walker B, and signature motif (Leu-Ser-Gly-Gly-Gln). After dimerization, the full transporter contains two nucleotide-binding sites (NBSs) that are formed by the Walker A (P loop), Q loop, and Walker B motifs from one protomer coupled with the signature motif from the other (10, 19, 20). In G5G8 only a single active NBS (NBS2), the one formed by the Walker A and B motifs of G5 and the signature motif of G8, is required to support sterol transport; scrambling the consensus motifs of the other NBS (NBS1) does not impair sterol export (21, 22).

Previously, we determined the X-ray structure of G5G8 in bicelles at 3.9-Å resolution (23). The transmembrane helices (TMHs) of G5G8 have a folding pattern that is distinct from all other families of ABC transporters that have been characterized structurally (10, 24). No structural information is available for G1 and the structure of ABCA1, although determined in apo states at ∼4-Å resolution (25), has not revealed the molecular basis of cholesterol transport. Notably, the structure of a multidrug exporter, ABCG2 (G2), in nanodiscs, revealed a sterol-like molecule bound to TMHs (26); however, there is no evidence to date that G2 can export sterols, so the function of the sterol in the TMHs remains unclear. Here, we report sterol-bound structures of G5G8 and apo-, cholesterol-, and ATP-bound structures of G1 by cryoelectron microscopy (cryo-EM). We have employed multiple strategies to establish a model of how ABC transporters mediate the translocation of neutral sterols across cell membranes.

## Sterol Binding Site 1 of G5G8

G5G8 exhibits pseudosymmetry, which complicates structural determination by cryo-EM. To break this pseudosymmetry and capture the sterol-bound state of G5G8 in solution, we developed a series of monoclonal antibodies that bind human G5 or G8 in its native state. We identified an antibody (2C7) that bound G8 and inhibited ATP hydrolysis by G5G8 in vitro (Fig. 1*C*). We then incubated the Fab fragments of 2C7 (Fab2C7) with purified G5G8 and subjected the mixture to gel filtration (*SI Appendix*, Fig. S1*A*).

The structure of G5G8 expressed in HEK293 cells in complex with Fab2C7 was determined at 2.7-Å resolution. The transporter is in an inward-facing conformation that is free of bound nucleotides, which is similar to that observed previously (23) (Fig. 1*D* and *SI Appendix*, Figs. S1 *B*–*E* and S2*A* and Table S1). Fab2C7 binds the nucleotide-binding domain (NBD) of G8 (*SI Appendix*, Fig. S2*B*) and restrains the conformational changes required for ATP hydrolysis. G5 and G8 share a similar conformation with an rmsd of 1.6 Å, but only G5 has an intramolecular disulfide bond (Cys587–Cys600) in its extracellular region (Fig. 1*E* and *SI Appendix*, Fig. S1*E*). TMH1, TMH2, and TMH5 of G5 and G8 form the interface between the two transmembrane domains (TMDs) (Fig. 1*E*). Connections between the NBDs and TMHs of G5G8 are formed by two three-helix bundles that include the connecting helix (CnH), coupling helix (CpH), and E helix (a helix after the Q loop). Both bundles in G5 and G8 contain two buried salt bridges, presumably to confine the TMHs and NBDs in a conformation conducive to engagement of substrate (Fig. 1*F*). The salt bridges between CpH and CnH of G8 had not been observed in the X-ray crystal structure (23).

**Fig. 2.**
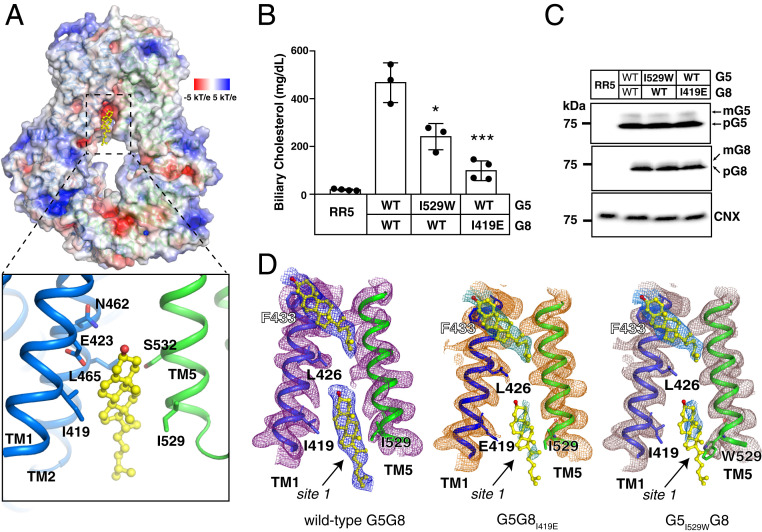
Overall structure of G5G8 reveals an inward-facing cholesterol-binding site. (*A*) Electrostatic surface representation of cholesterol-binding site 1 of G5G8 and the locations of interactions with cholesterol (yellow sticks). The residues that are involved in the interactions are shown as sticks. (*B*) Biliary cholesterol levels from *G5*^*−/−*^*G8*^*−/−*^ mice infected with recombinant adenoviruses expressing G5 and G8 (RR5 is an adenovirus without an insert). The mean ± SD of biliary cholesterol for each group of mice (*n* = 3 or 4 per group) is shown. *P < 0.05, ***P < 0.001, *t* test using GraphPad Prism 7. (*C*) Immunoblot analysis of G5G8 in liver membranes from adenovirus-treated mice. Calnexin served as an internal control. (*D*) Comparison of cholesterol maps in site 1 of wild-type G5G8 (*Left*), G5_I529W_G8 (*Middle*), and G5_I529W_G8 (*Right*). The maps are low-pass–filtered to 3.5-Å resolution and shown as mesh at 5σ level. The cryo-EM maps of the sterol-like molecule adjacent to residue F433 in the three transporters are similar whereas no sterol-like molecule is observed in site 1 of G5_I529W_G8 and G5G8_I419E_.

A sterol-like molecule was observed within the cytosolic leaflet, between the TMDs of G5 and G8, parallel to the TMHs (referred to as “site 1”) (Fig. 2*A*). The 3′-hydroxyl group of the sterol, which was presumed to be cholesterol, faces the central portion of the interface of G5 and G8, while the isooctyl side chain of cholesterol faces the cytoplasmic region of the protein (Fig. 2*A*). The cavity is large enough to accommodate other substrates of G5G8, such as campesterol or sitosterol (27), which both have a ring structure identical to that of cholesterol but have modifications in the side chains. Notably, the cavity has amphiphilic features (Fig. 2*A*), with the top being negatively charged and the bottom being hydrophobic. The cholesterol in site 1 is close to Ile529 in TMH5 of G5 and to Ile419 and Leu465 in TMH1 and TMH2 of G8, respectively.

The structure of G5G8 purified from *Pichia pastoris* was determined at 2.7-Å resolution (*SI Appendix*, Figs. S2 *C* and *D* and S3 and Table S1). A sterol-like density was observed in site 1, which we assigned to be ergosterol, the most abundant endogenous sterol in yeast. To validate site 1, we took advantage of an in vivo functional reconstitution assay that was established in our laboratory (28). Inactivation of G5G8 in mice (*G5*^*−/−*^*G8*^*−/−*^ mice) results in a marked reduction in the cholesterol content of bile (Fig. 2*B*). Biliary cholesterol secretion can be reconstituted by expressing recombinant G5 and G8 in *G5*^*−/−*^*G8*^*−/−*^ mice using adenoviral expression vectors. *G5*^*−/−*^*G8*^*−/−*^ mice were infected with adenoviruses expressing both wild-type G5 protein (G5_WT_) and G8_WT_ or mutant G5(WT) and G8(mutant) (or vice versa). After 3 d, bile was collected, and the sterol content was quantified using liquid chromatography–mass spectrometry. Expression of G5_WT_ and G8_WT_ resulted in a >20-fold increase in cholesterol content in bile (Fig. 2*B*). When we disrupted the hydrophobic character of site 1 by introducing the I419E mutation into G8 or when we substituted a bulky residue for residue Ile529 (I529W) in G5 to provide steric hindrance, biliary cholesterol levels decreased by over 50 to 80%. The levels of expression of both the precursor (pG5, pG8) and mature, fully glycosylated forms of G5G8 (mG5, mG8) were similar between all groups of mice (Fig. 2*C*). Thus, the mutations do not interfere with the folding, heterodimerization, or intracellular trafficking of G5G8 to the biliary membrane. Furthermore, we purified G5_I529W_G8 and G5G8_I419E_ from HEK293 cells and determined their structures at 3.5- and 3.1-Å resolution, respectively (*SI Appendix*, Fig. S4 and Table S2). Although the cryo-EM maps of the sterol-like molecule that is attached to the protein surface in the vicinity of residue Phe433 of G8 are similar to the WT protein, no sterol-like density is present in site 1 of G5_I529W_G8 and G5G8_I419E_ (Fig. 2*D*). These results confirm the important roles of G5-I529 and G8-I419 in substrate binding and provide further support for the premise that sterol binding to site 1 is required for sterol transport by G5G8.

## Sterol Binding Site 2 of G5G8

**Fig. 3.**
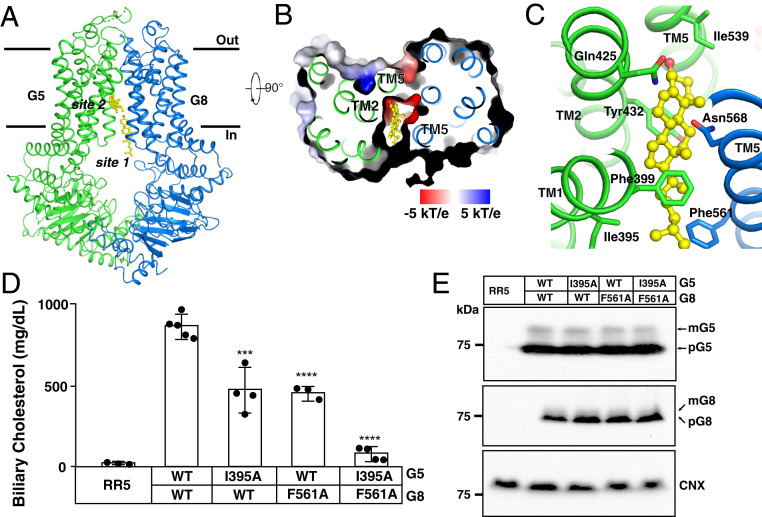
Cholesterol binding to a second site in G5G8. (*A*) Overall structure showing G5G8 bound to cholesterol (yellow sticks) viewed from the side of the membrane. (*B*) Electrostatic surface representation of G5G8 showing cholesterol bound to site 2. (*C*) The interaction details of cholesterol in site 2 with G5G8. The residues that are involved in binding cholesterol are shown as sticks. (*D*) Biliary cholesterol levels from *G5*^*−/−*^*G8*^*−/−*^ mice infected with adenoviruses expressing G5 and G8. The mean ± SD of the biliary cholesterol levels (*n* = 3 to 5 per group) for each experiment is shown. ***P < 0.001, ****P < 0.0001, *t* test using GraphPad Prism 7. (*E*) Immunoblot analysis of G5 and G8 expression in liver membranes from *G5*^*−/−*^*G8*^*−/−*^ mice expressing recombinant G5 and G8. CNX served as an internal control and was detected using an anti-CNX antibody.

To further dissect the molecular mechanisms of G5G8-mediated sterol export, we supplemented purified recombinant G5G8 expressed in yeast with cholesterol (0.5 mM) to saturate the sterol-binding sites. The structure of cholesterol-bound G5G8 was determined at 3.0-Å resolution (Fig. 3*A* and *SI Appendix*, Fig. S5 and Table S1). Two sterol molecules were identified: one in site 1 and the other buried in a more hydrophobic cavity located midway through the TMHs and oriented in a plane that was parallel to the membrane (referred to as “site 2”) (Fig. 3*B*). Since no sterol molecule was identified in site 2 without cholesterol supplementation (Fig. 2*A* and *SI Appendix*, Fig. S2*C*), we assigned a cholesterol molecule to this position. One part of site 2 is hydrophilic, comprising the side chains of Gln425 in TMH2, Ile539 of TMH5 in G5, and Asn568 in TMH5 of G8, while the other part is hydrophobic, formed by the Ile395 and Phe399 of TMH1 and Tyr432 of TMH2 in G5 and the residue Phe561 of G8-TMH5 (Fig. 3*C*).

To further validate that site 2 is a bona fide sterol-binding site, we performed more extensive mutagenesis. Substitution of G5-I395 and G8-F561 with alanine resulted in an ∼50% reduction in cholesterol export into bile. Substitution of both residues together resulted in almost complete inhibition of sterol transport (Fig. 3*D*). Neither of these mutations altered the expression or trafficking of G5 or G8 (Fig. 3*E*). Previously, we have found that substitution of alanine for tyrosine in position 432 of G5 (Fig. 3*C*) dramatically impaired cholesterol transport into bile (23). These experiments are consistent with the notion that site 2 plays a key role in cholesterol efflux.

## Structure of Cholesterol-Bound G1

**Fig. 4.**
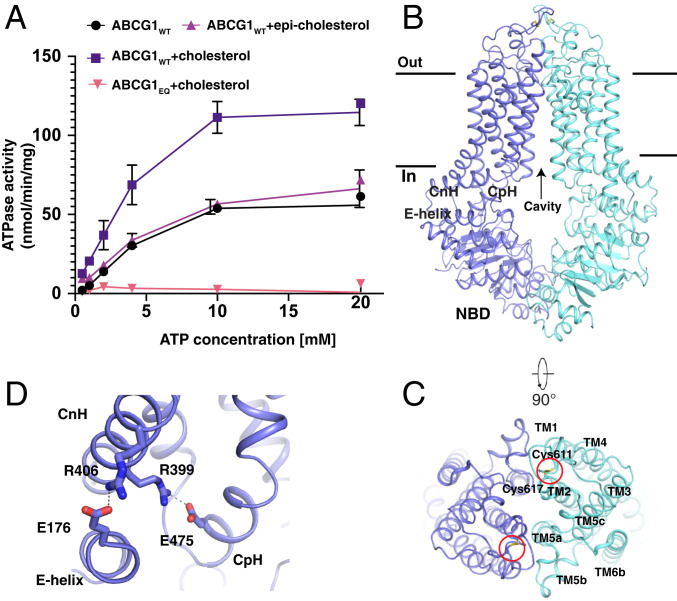
Overall structure of inward-facing G1 in the apo state. (*A*) Cholesterol stimulates the ATPase activity of G1_WT_. By nonlinear regression of the Michaelis–Menten equation, G1_WT_ has a *K*_m_ of 5.2 mM for ATP and a *V*_max_ of 153.3 nmol⋅mg^−1^⋅min^−1^ in the presence of 0.25 mM cholesterol; G1_WT_ has a *K*_m_ of 7.5 mM for ATP and a *V*_max_ of 82.6 nmol⋅mg^−1^⋅min^−1^ in the absence of cholesterol. Data are mean ± SD (*n* = 3 per group). (*B*) Overall structure of the apo G1_WT_ homodimer. The CpH, CnH, and E helix are indicated. (*C*) The extracellular view of TMHs. The disulfide bonds are shown by red circles. (*D*) The three-helix bundle of the CpH, CnH, and E helix. The residues are shown as sticks. The buried salt bridges are shown as dashed lines.

To further investigate how ABCG transporters mediate cholesterol efflux, we expressed G1_WT_ in *Sf9* cells and a catalytic mutant isoform E242Q (G1_EQ_) in HEK cells. The E242Q mutation abolishes ATP hydrolysis without interfering with ATP binding (29, 30). Basal ATPase activity of purified G1 was comparable to that seen for other ABC transporters (31–34). ATPase activity was stimulated by cholesterol but not its diastereomer, epi-cholesterol. This result is akin to other ABC transporters like G2 (33), ABCC1 (32), and NaAtm1 (35). As expected, no ATPase activity was detected for the catalytic mutant G1_EQ_ (Fig. 4*A*).

The structure of the G1_WT_ homodimer was determined at 3.7-Å resolution with C2 symmetry in an inward-facing conformation (Fig. 4 *B* and *C* and *SI Appendix*, Fig. S6 and Table S3). The overall fold of G1 is similar to that of G2 with an rmsd of 1.7 Å (*SI Appendix*, Fig. S7*A*) and G5G8 with an rmsd of 2.2 Å (*SI Appendix*, Fig. S7*B*), but very different from the folding pattern found in the A, B, and C subfamilies of ABC transporters, such as ABCA1 (25), P-glycoprotein (ABCB1) (36), and CFTR (ABCC7) (37) (*SI Appendix*, Fig. S7*C*). Residues Cys611 and Cys617 in the extracellular loop of each G1 protomer form an intramolecular disulfide bond (Fig. 4*C*). Similar disulfide bonds are present in the extracellular regions of G2 and G5, but not in G8. Unlike G2 (*SI Appendix*, Fig. S7*A*), G1 does not contain an intermolecular disulfide bond. The CnH, CpH, and E helix of each protomer form a three-helix bundle, as demonstrated previously in the other ABC transporters (Fig. 4*D*) (24). The transmembrane helices TMH2 and TMH5 of each G1 half-transporter form a hydrophobic cavity that is accessible to the cytosol (Fig. 4*B*).

**Fig. 5.**
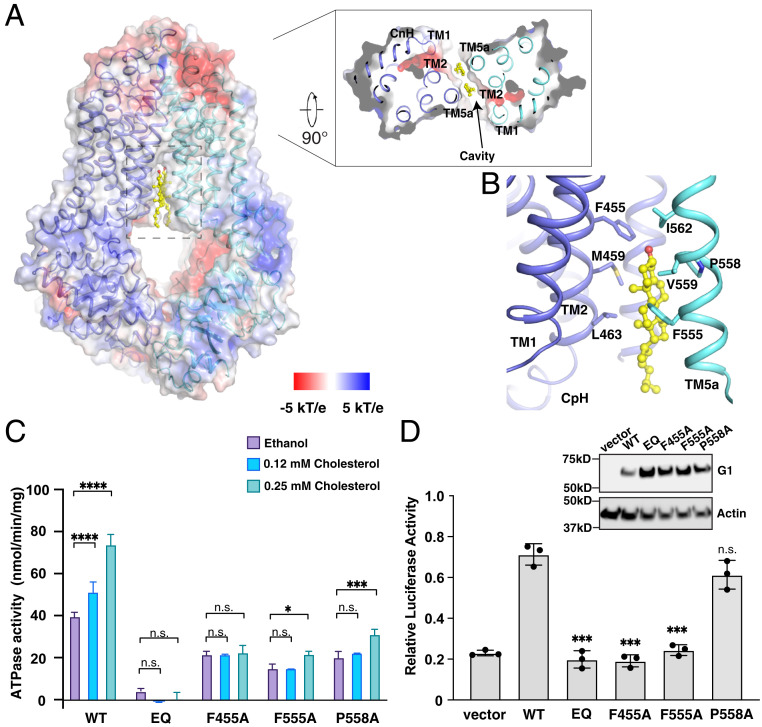
Overall structure of cholesterol-bound G1_EQ_. (*A*) The electrostatic surface representation of the cholesterol-binding cavity from membrane and cytosol. The cholesterol is shown as yellow sticks. (*B*) The interaction details between cholesterol and G1. The residues are shown as sticks. (*C*) The ATPase activity of purified G1 mutants in response to addition of cholesterol. The effect of cholesterol on ATPase activity was determined using a two-way ANOVA. (*D*) An SREBP-2/luciferase reporter assay to measure G1-mediated intracellular cholesterol transport activity. Western blot analysis of total cell extracts. Actin served as an internal control. *P < 0.05, ***P < 0.001, ****P < 0.0001; n.s., not significant. Data are mean ± SD (*n* = 3 per group).

We then determined the structure of G1_EQ_ after incubating the protein with cholesterol, ATP, and MgCl_2_ prior to grid preparation. Two states of G1_EQ_ were determined: One is inward-facing, similar to the conformation of G1_WT_ (no nucleotide bound to NBSs), and the other is outward-facing and has ATP bound to both NBSs (*SI Appendix*, Fig. S8). A sterol-like density was present in the hydrophobic cavity of the inward-facing G1_EQ_ (Fig. 5*A* and *SI Appendix*, Fig. S8*D* and Table S2). Since there is no notable density in the same position of G1_WT_, we assigned it as the cholesterol substrate. The residues Phe455, Met459, and Leu463 in TMH2 and residues Phe555, Pro558, Val559, and Ile562 in TMH5 of the other G1 molecule engage the putative cholesterol substrate (Fig. 5*B*). We tentatively built the 3′-hydroxyl group of cholesterol facing the center of G1, consistent with its orientation in G5G8 and G2 (Fig. 2*A* and *SI Appendix*, Fig. S9*A*) (26). The cholesterol-binding site in G2 accommodates a chemically similar modulator, thus preventing the binding of other substrates in the central site (*SI Appendix*, Fig. S9*B*) (38). We also observed a fatty acid chain-like density in the same cavity in cholesterol-bound G1_EQ_ (*SI Appendix*, Fig. S9*C*); however, the identification and function of this molecule remain unclear.

To validate the roles of key residues in cholesterol recognition, we assayed the ATPase activities of purified G1 mutants expressed in *Sf9* cells to determine whether their activity is stimulated by cholesterol. Each mutant showed a similar behavior in solution during purification as G1_WT_ (*SI Appendix*, Fig. S10). The results showed that mutations in the cholesterol-binding site, including F455A, F555A, and P558A, decrease the basal activity of G1 (Fig. 5*C*), which is consistent with the finding that some mutations in the substrate-binding site of G2 also decrease basal ATPase activity (33, 39). Unlike G1_WT_, the ATPase activities of G1_F455A_ and G1_F555A_ are not enhanced by cholesterol. A significant increase in ATPase activity was seen only in G1_P558A_ upon incubation of the protein with 0.25 mM cholesterol (Fig. 5*C*).

To assess the effects of these mutations on G1-mediated sterol transport, we took advantage of an established assay that monitors the effect of G1 expression on cells: G1 expression in cells results in a redistribution of intracellular cholesterol and activation of the cholesterol-regulated transcription factor sterol regulatory element binding protein (SREBP)-2 (40). We used an SREBP-dependent luciferase assay to monitor the intracellular cholesterol export activity of G1. Luciferase activity of cells expressing G1_WT_ was threefold higher than cells that were transfected with the empty vector, or with G1_EQ_, G1_F455A_, and G1_F555A_ (Fig. 5*D*). The luciferase activity of cells expressing G1_P558A_ decreased slightly; this result is consistent with the findings that the ATPase activity of G1_P558A_ is stimulated with addition of cholesterol in an in vitro assay (Fig. 5*C*).

## Structure of ATP-Bound G1

**Fig. 6.**
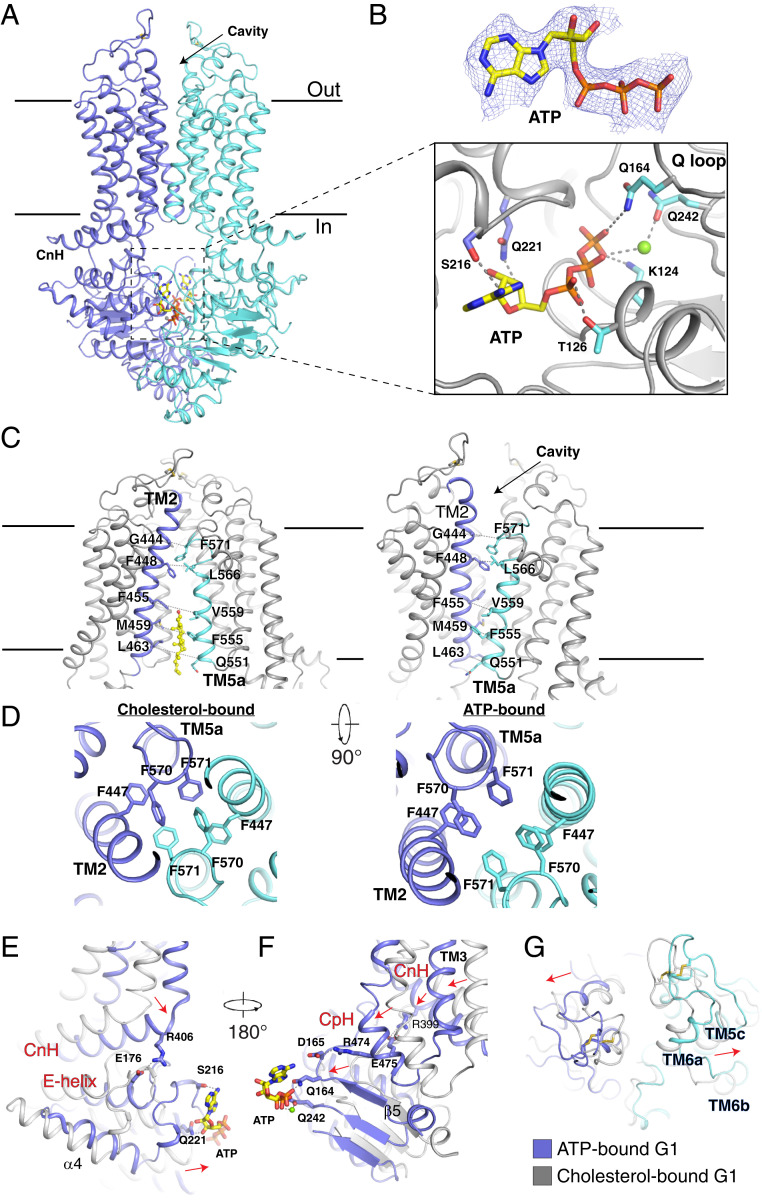
Overall structure of ATP-bound G1_EQ_. (*A*) Overall structure of ATP-bound G1_EQ_. The cryo-EM map of ATP is shown at 5σ level. (*B*) The interaction details between ATP and G1_EQ_. The residues and ATP are shown in stick representation and the putative Mg^2+^ is shown as a green sphere. (*C*) The structural comparison of inward-facing (*Left*) and outward-facing (*Right*) G1_EQ_. The distance between the Cαs of specific residues is shown as dashed lines. (*D*) Phenylalanine residues in TMDs regulate the accessibility of the channel in G1. (*E* and *F*) ATP binding–induced conformational changes in CnH and CpH. The structural movements after ATP binding are indicated by red arrows. (*G*) Structural comparison of the extracellular loops in cholesterol-bound and ATP-bound G1. The disulfide bonds are indicated by yellow sticks in *A*, *C*, and *G*.

The structure of ATP-bound, outward-facing G1_EQ_ was determined at 3.7-Å resolution (Fig. 6*A* and *SI Appendix*, Fig. S8 *F*–*H* and Table S3). Two ATP molecules were identified in the cryo-EM map. Residues Lys124, Thr126, Gln164, and Gln242 (Glu242 in G1_WT_) plus residues Ser216 and Gln221 of the contralateral G1_EQ_ interact with ATP (Fig. 6*B*). In the ATP-bound form of G1, the cavity in the cytosolic leaflet is closed and another cavity appears in the extracellular leaflet (Fig. 6*C*). The distances between Cα of Leu463 and Gln551 change from 12 to 7 Å, while the distances between Cα of Gly444 and Phe571 increase by ∼2 Å to open the extracellular cavity (Fig. 6*C*). In the cholesterol-bound state, Phe447 in TMH2 and Phe570 and Phe571 in the C terminus of TM5Ha pack together through π–π interactions with the same residues of the contralateral G1 to form a plug that blocks the central conduit.

When ATP binds the NBS, the residues move ∼4 Å away from the core, opening an extracellular cavity (Fig. 6*D*). After binding ATP, the E helix of G1 is in the center of the protein, causing a 6-Å shift of the CnH (Fig. 6*E*). Residue Gln164 triggers the movement of β5, inducing an ∼3-Å shift of the CpH, which in turn results in movement of TMH3 into the center (Fig. 6*F*). These changes prompt TMH5a to move toward the center, thus closing the cavity in the cytosolic leaflet (Fig. 6*D*). ATP binding induces the extracellular regions of G1 to move 4 Å away from the center to the edge of the transporter (Fig. 6*G*).

## Structural Comparison of G1, G5G8, G2, and ABCA1

**Fig. 7.**
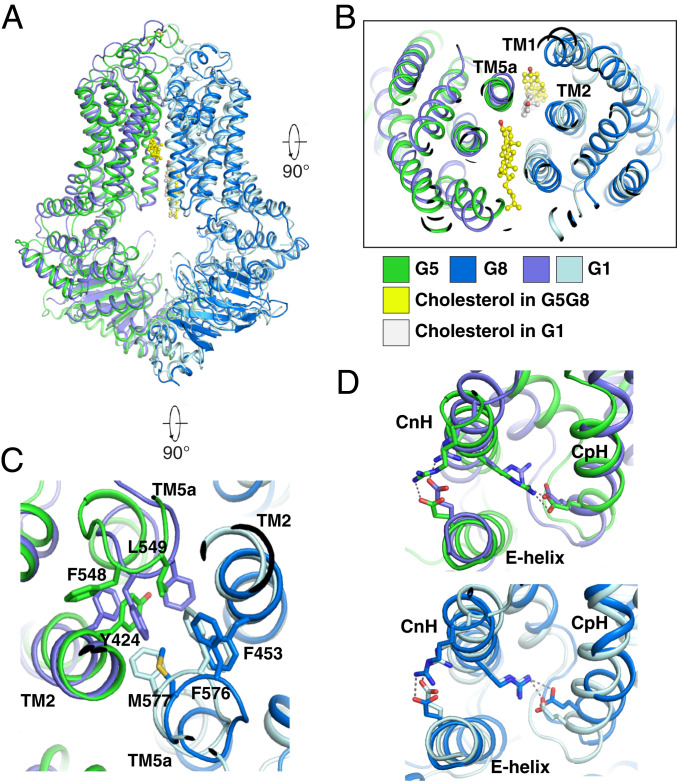
Structural comparison of cholesterol-bound G5G8 with G1. (*A*) Overall structural comparison of cholesterol-bound G5G8 with cholesterol-bound G1_EQ_. (*B*) The extracellular view showing that TM1 of G8 is involved in binding cholesterol. (*C*) The structural comparison of the core residues in G5G8 with that in cholesterol-bound G1_EQ_. Aromatic and hydrophobic residues in TMDs of G5G8 are indicated; the view and residues in G1 are consistent with that in Fig. 6*D*. (*D*) Structural comparison of buried salt bridges in cholesterol-bound G5G8 and G1_EQ_. The three-helix bundle is labeled, and the salt bridges are indicated by dashed lines.

Sterol-binding site 1 of G5G8 is similar in position to that of G1 (Fig. 7*A*). Taken together with the finding of a sterol-like lipid or detergent in the cytosolic leaflet of ABCA1 (*SI Appendix*, Fig. S7*C*), it is tempting to speculate that the three cholesterol exporters are loaded with cholesterol in a similar fashion. In G1, TM1 does not engage the sterol substrate. In G5G8, TM1 of G8, but not of G5, engages the sterol substrate (Fig. 7*B*). Notably, there is no substrate-binding site in G2 that is equivalent to site 1 in G1 or G5G8 (*SI Appendix*, Fig. S9 *B* and *D*), presumably because G2 transports hydrophilic molecules that can access G2 directly from the cytosol. In contrast, neutral sterols require a binding site that is accessible to the cytosolic leaflet of the membrane so that it can physically engage with the transporter without contacting water or hydrophilic molecules. Although cholesterol can flip spontaneously between the inner and outer leaflets of membranes, even in the absence of proteins (2), it is not clear how the cholesterol in the bilayer would enter site 1. Alternatively, a carrier protein may deliver the sterol into the sterol-binding site. Like G2 and G5G8, G1 and ABCA1 might have a central cavity that serves as a midpoint along the cholesterol efflux pathway, although no such sites have been captured in the structural models available to date (*SI Appendix*, Fig. S9 *D*–*F*). In G5G8, the 3′-hydroxyl group of the sterol substrate is oriented toward the hydrophilic regions of sites 1 and 2 (Figs. 2*A*, 3*B*, and 7*B*). Sterols in site 1 may pivot to the midpoint of the translocation pathway (site 2) without flipping during the export process.

G5G8 and G1 share structural features in common, suggesting that these two transporters translocate sterols across membranes using similar mechanisms. First, they both have similarly positioned aromatic plugs, which in G1 modulates the core size of the cholesterol translocation pathway (Figs. 6*D* and 7*C*). Second, they have four buried salt bridges among the CnH–CpH–E helix bundles, which are required for coupling the energy generated from ATP hydrolysis to the conformational changes of TMDs (Fig. 7*D*).

## Unique Features of G8

**Fig. 8.**
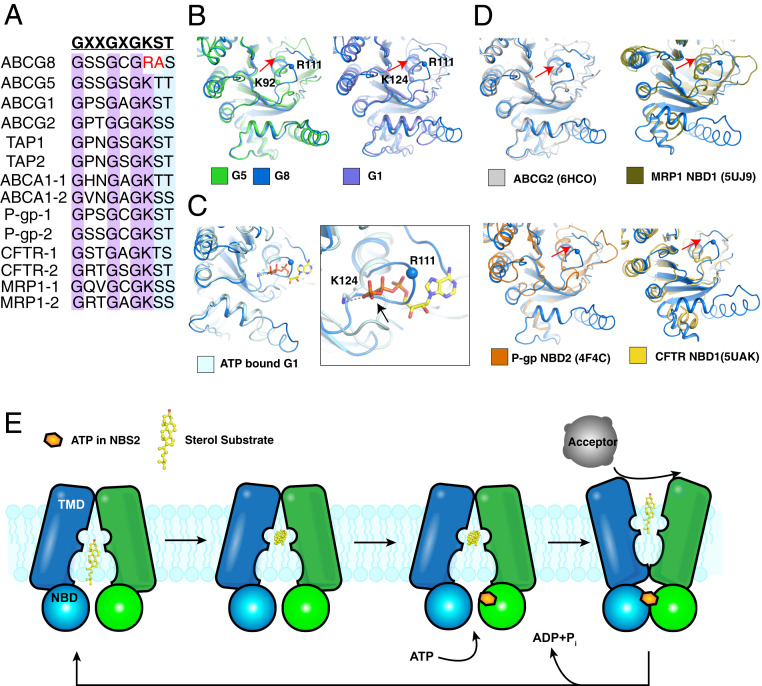
Unique feature of the G8 P loop and a model for G5G8-mediated sterol export. (*A*) The sequence alignment of the P loop of human ABC transporters. The conserved residues are highlighted. (*B*) Structural comparisons of G8-NBD with that of G1 and G5. (*C*) The P loop of G8 interferes with ATP binding. The potential steric clash is indicated by a black arrow. (*D*) Structural comparisons of G8-NBD with that of other ABC transporters. The movement of the G8 P loop is indicated by red arrows. R111 of G8 is shown as a ball, as well as the crucial lysine of other NBDs. (*E*) A proposed model of G5G8. The cytosolic leaflet cavity engages sterols that further traffic from this site halfway through the TMHs. The conformation becomes closed when the ATP is engaged by NBS2 and pushes the cholesterol substrate to extracellular lipid acceptors. After ATP hydrolysis, the transporter returns to the resting state.

Our previous study revealed a striking asymmetry in the ATPase activities of the two NBSs: Only the Walker A (P loop) and Walker B motifs of G5 and the signature motif of G8 are required for ATP-driven cholesterol export in vivo (22). Notably, a conserved lysine in the P loop, which is essential for engaging ATP (Lys124 of G1 and Lys92 of G5), is substituted by an arginine (Arg111) in G8. This mutation is not present in the degenerate sites of any of the other mammalian ABC transporters that have been sequenced (Fig. 8*A*).

The P loop of G8, which is located in NBS1 of G5G8, is shifted laterally by ∼10 Å relative to the P loops of G5 or G1. This shift is predicted to introduce a steric hindrance for ATP binding (Fig. 8 *B* and *C*). A broader comparison of this degenerate ATPase with other ABC transporters supports the notion that the change seen in NBS1 of G5G8 is distinct (Fig. 8*D*). Of the other 29 full-length or heterodimeric ABC transporters in the mammalian genome, 21 have one active and one degenerate NBD. In almost all cases, the glycine in the signature sequence is mutated or the “catalytic” glutamate in the Walker B motif is changed to aspartate (e.g., TAP1 and MRP1) or serine (e.g., CFTR) (41). These changes do not alter the overall structure of the NBS; ATP binding is retained but ATP hydrolysis is abolished (37). The changes are highly conserved in these transporters, indicating ongoing evolutionary pressure on the sequence of the degenerate NBD. In contrast, the sequence change in the P loop of the degenerate NBS of G5G8 dramatically alters the fold (Fig. 6*B*) and results in a marked decrease in ATP binding (22).

## Cholesterol Transport by G1 and G5G8

How do rigid hydrophobic sterols get exported across the membrane by these two ABC transporters? Molecular dynamics simulations predicted that within 100 ns of simulation, sterol leaves site 2 of G5G8 in an orientation such that the 3′-hydroxyl group faces the extracellular space (Movie S1). When the transporter is in the inward-facing conformation, a cavity in the cytosolic leaflet engages the sterol substrate (site 1). The substrate may traffic from site 1 to the more hydrophobic site 2 in the center of the TMHs (Figs. 3*A* and 8*E*). It remains unclear how sterol trafficking in the channel in the TMHs is related to ATP binding or hydrolysis. G1 assumes a closed conformation when ATP binds the NBS, which collapses site 1 while simultaneously pushing the neutral sterol substrate to the extracellular cavity, where it binds extracellular lipid acceptors or enters the outer hemileaflet of the bilayer. Upon extracellular cholesterol release and ATP hydrolysis, the transporter returns to the resting state, ready to transport another cholesterol molecule (35, 42–44). Further structural analysis of G5G8 will be required to determine how ATP hydrolysis can rearrange the TMHs so that it flips from an inward- to an outward-facing state, as has been found to occur in other ABC transporters.

## Materials and Methods

### Generation of Anti-Human G5G8 Antibody.

Immunoglobulin G (IgG)-2C7, a mouse monoclonal anti-human G5G8 antibody, was prepared by fusion of SP2-mIL6 mouse myeloma cells with splenic B lymphocytes obtained from BALB/c mice (*n* = 2). Mice were immunized with one primary and eight boosts of purified recombinant human G5G8 heterodimers (50 μg) in 10 mM Hepes (pH 7.5), 100 mM NaCl, 0.1% n-dodecyl-β-D-maltopyranoside (DDM), 0.05% cholate, and 0.1 mM tris(2-carboxyethyl)phosphine (TCEP) combined with the Sigma Adjuvant System. Hybridoma culture supernatants were screened by enzyme-linked immunosorbent assay (ELISA) and counterscreened by dot blot to select ELISA-positive, dot blot–negative clones. One such hybridoma, designated IgG-2C7 (subclass 1, k), was subcloned by serial dilution three times and purified from hybridoma culture supernatant by gravity-flow affinity chromatography on protein G Sepharose 4 Fast Flow columns.

### Protein Expression and Purification.

The complementary DNA (cDNA) of human ABCG1 (GenBank accession no. BC029158.1) was cloned into pFastBac with an N-terminal Flag tag. The G1_WT_ protein was expressed using baculovirus-mediated transduction of *Sf9* insect cells (ATCC). At 48 h post infection, the cells were disrupted by sonication in buffer A, containing 20 mM Hepes (pH 7.5), 150 mM NaCl, with 1 mM phenylmethanesulfonylfluoride and 5 μg/mL leupeptin. After low-speed centrifugation, the resulting supernatant was incubated in buffer B with 1% (weight/volume; wt/vol) lauryl maltose neopentyl glycol (LMNG; Anatrace) for 1 h at 4 °C. The lysate was centrifuged at 18,000 rpm for 30 min, and the supernatant was loaded onto a Flag-M2 affinity column (Sigma-Aldrich). After washing three times, the protein was eluted in 20 mM Hepes (pH 7.5), 150 mM NaCl, 100 μg/mL 3×Flag peptide, and 0.01% LMNG and concentrated. The concentrated protein was purified by a Superose 6 Increase size-exclusion chromatography column (GE Healthcare) in a buffer containing buffer A and 0.06% (wt/vol) digitonin (ACROS Organics). The cDNA of the E242Q mutant was generated using the primers 5′-AGT​CAT​GTT​CTT​CGA​TCA​GCC​CAC​CAG​CGG​CCT-3′ and 5′-AGG​CCG​CTG​GTG​GGC​TGA​TCG​AAG​AAC​ATG​ACT-3′ and cloned into pEG BacMam with an N-terminal Flag tag. The protein was expressed using baculovirus-mediated transduction of mammalian HEK-293S GnTI^−^ cells (ATCC). The cells were harvested at 48 h post infection and the protein was purified the same as G1_WT_.

The cloning and expression of recombinant human G5G8 in *P. pastoris* were performed as described previously (23). The expression of human G5G8 in mammalian HEK-293S cells was performed by cloning the cDNAs for human ABCG5 (National Center for Biotechnology Information [NCBI] accession no. NM_022436) and ABCG8 (NCBI accession no. NM_022437) into separate pEG BacMam, respectively, and they were coexpressed using baculovirus-mediated transduction of mammalian HEK-293S GnTI^−^ cells (ATCC). A tandem tag of six histidines separated by glycine (His_6_GlyHis_6_) was added to the C terminus of G5, and a tag encoding a rhinovirus 3C protease site followed by a calmodulin-binding peptide was added to the C terminus of G8, for purification purposes. At 72 h post infection, the cells were collected by centrifugation and the recombinant protein was solubilized and purified as previously described (23). The expressed human G5G8 was purified as described (23) except that cholesteryl hemisuccinate Tris was not added to any buffer, and one more purification step was added using Superdex 200 Increase 10/300 GL for gel filtration with a buffer containing 20 mM Hepes (pH 7.5), 150 mM NaCl, 2 mM MgCl_2_, 2 mM ATP, and 0.06% (wt/vol) digitonin (Calbiochem).

### ATPase Assays.

The ATPase activity of purified G5G8 was determined as described (23, 45). Briefly, 4 to 10 µg proteins was mixed with 100 µg liver polar lipids (Avanti), 5 mM dithiothreitol (DTT), and 1% sodium cholate for 10 min at room temperature. Reactions were carried out in a final volume of 100 μL containing 50 mM Tris⋅HCl (pH 7.5), 60 mM NaCl, 30 mM KCl, 2.5 mM MgCl_2_, and 2.5 mM γ-[^32^P]ATP at 37 °C for 30 min. Released inorganic [^32^P]phosphate was extracted by molybdate and the radioactivity was measured to calculate its specific activity in three independent experiments.

The ATPase activity of G1 was measured using an NADH consumption-coupled method (46, 47). The assay was performed at 37 °C in a 96-well plate with a total reaction volume of 100 μL. Absorbance at 340 nm was monitored to measure the concentration of NADH which was coupled to that of ATP. The final reaction included 0.3 to 0.5 μM G1, 0.2 mM NADH, 4 mM phosphoenolpyruvate, 60 μg/mL pyruvate kinase, 33 μg/mL lactate dehydrogenase, 1 mM DTT, 2 mM MgCl_2_, 0.06% digitonin, 150 mM NaCl, and 20 mM Hepes (pH 7.5). For the ATP titration assay (Fig. 4*A*), 0.5 to 20 mM ATP was included in the final reaction, with the presence of 0.25 mM cholesterol and 0.25 mM epi-cholesterol or an equal volume of ethanol. For measuring the effect of cholesterol ATPase activity (Fig. 5*C*), 8 mM ATP and 0.5 μM G1 protein were supplemented across all reactions. Due to its instability, NADH was dissolved and added right before the start of the reaction. The plate and reaction stock solution were prewarmed before the different reacting components were mixed. The absorbance was measured every 20 s for 60 min using a BioTek Synergy Neo plate reader. The *V*_max_ of absorbance change (min^−1^) was calculated by the built-in software using 20 to 30 points in the linear region, which was converted to the rate of ATP hydrolysis (nmol ATP⋅min^−1^⋅mg protein^−1^) by dividing the product of the extinction coefficient of NADH, the length of the light path, and the concentration of G1. An identical reaction containing buffer instead of G1 protein was measured in the same plate and was deducted from the corresponding experimental group.

### In Vivo Functional Reconstitution Cholesterol Transport Assay.

Point mutations were introduced into the human G5 and G8 cDNAs using the QuikChange II Site-Directed Mutagenesis Kit (Agilent). The recombinant adenoviruses expressing human WT or mutant were generated using the AdenoVator Adenoviral Vector System (QBioGene). Eight- to 12-wk-old total knockout (*G5*^*−/−*^*G8*^*−/−*^) mice were maintained on a regular chow diet (48). Adenoviral particles (5 × 10^12^ particles per kilogram), containing no external gene (RR5) or WT or mutant human G5G8, were injected into the tail veins of the mice. After 72 h, the mice were fasted for 4 h, anesthetized with halothane, and killed by exsanguination. Bile was collected, and neutral sterol levels were measured using gas liquid chromatography and mass spectrometry as described (23). Liver tissue was snap-frozen in liquid nitrogen and stored at −80 °C. All animal experiments described in this manuscript were approved and conducted under oversight of the UT Southwestern Institutional Animal Care and Use Committee.

### Immunoblot Analysis of Expression of G5G8 in Mouse Liver and G1 in CHO-K1 Cells.

Mouse livers were cut into small pieces, washed with ice-cold buffer containing 0.2 M sucrose, 50 mM Tris-4-morpholineethanesulfonic acid (MES) (pH 7.0), and 0.1 M NaCl, and homogenized in a 3× volume of buffer. The homogenate was centrifuged at 1,500 × *g* for 10 min. The resulting postnuclear membrane was centrifuged at 100,000 rpm in a TLA100.4 rotor for 15 min at 4 °C. The pellets were resuspended in the same buffer and protein concentration was measured. For Western blot, 25 µg protein of pooled membranes for each group of samples was used for each lane. Antibodies (Abs) used were as follows: for human G5: monoclonal Ab 13H11, 10 µg/mL (made in-house); for human G8: monoclonal Ab 8E3, 10 µg/mL (made in-house); and for calnexin (CNX), polyclonal Ab (Enzo; ADI-SPA-860-F), 4,000× dilution.

To detect expression of G1 in CHO-K1 cells, a total of 2.5 × 10^5^ cells were resuspended in RIPA buffer. After a high-speed centrifugation, the supernatant was incubated with solubilization buffer (62 mM Tris⋅HCl, pH 6.9, 15% sodium dodecyl sulfate, 8 M urea, 10% glycerol, and 100 mM DTT, at a 1:1 volume ratio) at 37 °C for 30 min. After electrophoresis the proteins were transferred to nitrocellulose filters. The filters were incubated with anti-G1 rabbit polyclonal antibodies (1:500; Novus Biologicals; NB400-132) at 4 °C overnight, followed by horseradish peroxidase (HRP)–linked anti-rabbit IgG (1:5,000; Cell Signaling Technology) at room temperature for 30 min. HRP-conjugated β-actin antibody (1:5,000; Cell Signaling Technology) was used to visualize the proteins using a SuperSignal West Pico PLUS Chemiluminescent Substrate Kit (Thermo Fisher Scientific). Images were scanned and analyzed using an Odyssey Fc Imaging System (LI-COR Biosciences).

### Luciferase Reporter Assay.

The cDNA of human ABCG1 was cloned into pcDNA3.1 without a tag. Cells were transfected using FuGENE HD (Promega) according to the manufacturer’s instructions. On day 0, CHO-K1 cells were maintained in medium A, a 1:1 mixture of Ham’s F-12 medium and Dulbecco’s modified Eagle’s medium (DMEM) containing 2.5 mM l-glutamine,100 U/mL penicillin, 100 μg/mL streptomycin sulfate, and 5% fetal calf serum (FCS) at a density of 8 × 10^4^ cells per well on 24-well plates. On day 1, monolayers were replaced with medium A and each well was transfected with 100 ng pSynSRE (Addgene), plus 5 ng of each expression plasmid, and 50 ng pRL-TK (Promega) as a control to normalize for changes in transfection efficiency according to a previously published protocol (40). After 5 h, the culture medium was switched to medium A with 10% FCS. On day 3, after being cultured for 22 h, cells were washed with phosphate-buffered saline. Firefly and *Renilla* luciferase activities were measured using the Dual-Luciferase Reporter Assay System (Promega). The data analysis was performed using Prism 7 (GraphPad Software). Results are shown as mean ± SD from three biologically independent experiments.

### EM Imaging Processing, Three-Dimensional Refinement, and Molecular Dynamics Simulation.

The details are in *SI Appendix*.

### Reproducibility.

All animal experiments were repeated at least two times on different days. All cell biological and biochemical experiments were repeated at least three times on different days. Similar results were obtained.

## Supplementary Material

Supplementary File

Supplementary File

## Data Availability

The three-dimensional cryo-EM density maps reported in this article have been deposited in the Electron Microscopy Data Bank under the accession nos. EMD-24315 (G1_WT_), EMD-24316 (cholesterol-bound G1_EQ_), EMD-24317 (ATP-bound G1_EQ_), EMD-24313 (HEK-expressed G5G8), EMD-24311 (HEK-expressed G5_I529W_G8), EMD-24310 (HEK-expressed G5G8_I419E_), EMD-24312 (yeast-expressed G5G8), and EMD-24314 (cholesterol-bound G5G8). Atomic coordinates for the atomic model have been deposited in the Protein Data Bank under ID codes 7R8C (G1_WT_), 7R8D (cholesterol-bound G1_EQ_), 7R8E (ATP-bound G1_EQ_), 7R8A (HEK-expressed G5G8), 7R88 (HEK-expressed G5_I529W_G8), 7R87 (HEK-expressed G5G8_I419E_), 7R89 (yeast-expressed G5G8), and 7R8B (cholesterol-bound G5G8). All study data are included in the article and/or supporting information.
