# Supplementary material for: Molecular basis of cholesterol efflux via ABCG subfamily transporters
Source: Proc Natl Acad Sci U S A. 2021 Aug 17;118(34):e2110483118. doi: 10.1073/pnas.2110483118 (PMC8403869; doi:10.1073/pnas.2110483118)
Supplement: Supplementary File [file pnas.2110483118.sapp.pdf]

PNAS
www.pnas.org

Supplementary Information for

Molecular basis of cholesterol efflux via ABCG subfamily transporters

Yingyuan Sun, Jin Wang, Tao Long, Xiaofeng Qi, Linda Donnelly, Nadia Elghobashi-Meinhardt,

Leticia Esparza, Jonathan C Cohen, Xiao-Song Xie, Helen H Hobbs* and Xiaochun Li*

*Correspondence: Helen.Hobbs@UTSouthwestern.edu (H.H.H.) or

Xiaochun.Li@utsouthwestern.edu (X.L.).

This PDF file includes:

Supplementary text
Figures S1 to S10
Tables S1 to S3
Legends for Movie S1
SI References

Other supplementary materials for this manuscript include the following:

Movie S1

Supporting Text

Methods

EM Sample Preparation and Imaging

For $G1_{WT}$ and $G1_{EQ}$, 8 mM ATP, 8 mM $MgCl_2$ and 0.5 mM cholesterol (dissolved in ethanol) was added into 8 mg/ml protein and the mixture was incubated at room temperature for 20 minutes. For G5G8, yeast- or HEK- expressed G5G8 protein, with or without sterols, complexed with Fab 2C7 was concentrated to ~15mg/ml. Aliquots of 3 μl of the sample were loaded onto Quantifoil R1.2/1.3 400 mesh Au holey carbon grids (Quantifoil), blotted using a Vitrobot Mark IV (FEI), and plunged into liquid ethane for flash freezing. The grids were imaged in a 300 keV Titan Krios (FEI) with a Gatan K3 Summit direct electron detector (Gatan). Data were collected at 0.833 Å/pixel (UTSW), 0.844 Å/pixel or 0.832 Å/pixel (Janelia Research Farm) with a dose rate of 23 electrons per physical pixel per second. Images were recorded for 1.8 s exposures in 60 subframes to give a total dose of 60 electrons per $Å^2$.

Imaging Processing and 3D Reconstruction

For the $G1_{WT}$ dataset, 5,435 movies were collected. Dark subtracted images were first normalized by gain reference and correction of the beam-induced motion was performed using the program MotionCor2 (49). The contrast transfer function (CTF) was estimated using CTFFIND4 (50). Images that had ice contamination or bad CTF Thon rings were discarded and 4,810 images were left. About 1500 particles were manually picked and classified by 2D classification in RELION-3 (51). The 2D class averages were used as the templates for auto-picking in RELION-3. About 1 million particles were picked and

extracted. After 2D classification using cryosparc (52), 900k particles were kept. We used the cryo-EM structure of $G1_{EQ}$ as the initial model with a C1 symmetry for 3D classification. The best class, containing 545,470 particles, was selected and used for subsequent 3D auto-refinement in RELION-3 with C2 symmetry which yielded a reconstruction of 4.1 Å. The refined particles were polished and subject to a second round of 3D refinement which improved the resolution to 3.88 Å. At last, one round of CTF refinement was performed and yielded the final map at 3.73 Å (with mask). Resolution was estimated using the Fourier shell correlation (FSC) 0.143 criterion.

For the $G1_{EQ}$ dataset, 4,953 movies were collected. A total of 1.44 million particles were auto-picked and 1.35 million were kept after 2D classification. A 3D map from an Arctica Talos 200kV session was used as the initial model for 3D classification and resulted a good class with 1.02 million particles. An initial 3D refinement yielded a reconstruction at 3.8 Å which appeared as the "inward-facing" conformation. A subsequent 3D classification without image alignment resulted in 5 classes. The major class, containing 408,553 particles, is in a substrate-bound, inward-facing conformation. After particles polishing, and CTF refinement, a final map at 3.23 Å was achieved and interpreted as the $G1_{EQ}$ cholesterol-bound state. Another class, containing only 111,370 particles, appeared as a more open conformation and the NBDs were much closer to each other. After particle polishing, a reconstruction at 3.88 Å was achieved. To further reduce structural heterogeneity, a second round of 3D classification without alignment was performed and 72,323 particles were kept which were subjected to a round of CTF

refinement. The final reconstruction resulted in a map at 3.7 Å (with mask) and represented an ATP-bound state.

The overall data processing procedures are the same for the five G5G8 datasets. Here we describe the data processing of the yeast expressed G5G8 without additional sterols as an example. For this dataset, 10,235 movies were collected. Beam-induced motion between the frames was corrected using the program MotionCor2 (49). The contrast transfer function (CTF) was estimated using CTFFIND4 (50). About 1,800 particles were manually picked and classified by 2D classification in RELION-3 (51). The 2D class averages were used as the templates for auto-picking in RELION-3 and over 1.7 million particles were picked and extracted. Approximately 1.4 million particles were kept after 2D classification. A map reconstructed from a previous 200kV dataset was used as the initial model for 3D classification. 574,697 particles were selected and refined in RELION-3 with C1 symmetry which yielded a reconstruction of 3.7 Å. The refined particles were polished and subject to a round of CTF refinement after which the resolution was improved to 2.7 Å. A second round of 3D classification without alignment was performed and improved the resolution to 2.6 Å with 471,984 particles. The final map was post-processed in RELION-3 with a B-factor of 30 $Å^2$. Resolution was estimated using the Fourier shell correlation (FSC) 0.143 criterion with mask applied. More details of the data processing can be found in Figs. S1, S3, S4, S5, S7 and S9.

Model Construction

To obtain better side-chain densities for model building, we sharpened the maps using post-processing in RELION-3. For the $G1_{WT}$, the initial model was built using SWISS-MODEL (53) with ABCG2 (54) as the template. The positions of the individual residues were adjusted using COOT (55). In all three G1 maps, the densities of N-terminus (residues 1-71), the loop between the β1 and β2 (residues 87-100) and the loop before the CnH (residues 348-384) were not resolved nor built. For G5G8, PDB-5DO7 was used as a reference for the initial assignment of the secondary elements. The relatively high resolution of the maps allows accurate positioning of most of the amino acids in both the transporter and the Fab. Due to flexibility, residues 47-65, 103-106, 589-596 of G5 and residues 57-86, 123-125, 208-209, 326-391, 612-625 of G8 were not built (these regions vary slightly in three structures). The variable region of the Fab 2C7 was built whereas the constant region was not built due to the lack of good density.

Model Refinement and Validation

The models were refined in real space using PHENIX (56) and also in reciprocal space using Refmac with secondary-structure restraints and stereochemical restraints (57). The model vs. map FSC curves were generated in the comprehensive validation module in PHENIX. PHENIX and MolProbity (58) were used to validate the final model. Local resolutions were estimated using RELION-3. Structure figures were generated using PyMOL (http://www.pymol.org) or Chimera (59).

Construction of Model for Simulation

The unassigned internal residues of G5G8 in the model were reconstructed manually. Hydrogen atoms were added using H-build from CHARMM (60). An initial protonation pattern was obtained by calculating the pKa values of all titratable residues. For this, electrostatic energy computations were carried out with karlsberg+ (61). Based on these results, a model was built to represent the protonation pattern at pH 7. This model included the following non-standard protonated amino acid sidechains: Glu326 on G5, and Asp275, His420, and Asp466 on G8. To position the TMHs in the membrane, the previously determined structure (PDB:5DO7) was used as a template. The two structures were overlapped using Chimera and the new protein coordinates were saved. Next, using the CHARMM-GUI (62) and OPM database (63), the protein was inserted in a lipid bilayer consisting of an cytosolic leaflet and an extracellular leaflet. Both leaflets contain 40% cholesterol. In the cytosolic leaflet, the remainder is comprised of 20% dilauroyl-D-glycero-1-Phosphatidylethanolamine (DLPE), 20% DLPS (dilauroyl-D-glycero-1-Phosphatidylserine), and 20% Di-myristoyl-inositol (DMPI); the extracellular leaflet contains 30% sphingomyelin and 30% didecanoyl-D-glycero-3-Phosphatidylcholine (DDPC). The protein-membrane system was next solvated in water with 0.15 M NaCl concentration, resulting in Na^+ (226) and Cl^- (150) ions to neutralize charge, and 54,015 explicit TIP3 water molecules (64). The total system consists of 231,274 atoms and was simulated in a rectangular box of dimension 125.6Å x 125.6Å x 158.9Å.

Geometry Optimizations and Molecular Dynamics

The initial geometry of each solvated protein-membrane complex was optimized with 1500 steps of steepest descent (SD) energy minimization, followed by 1500 adopted basis

Newton-Raphson (ABNR) (60) steps to remove any close contacts. All energy minimizations and geometry optimizations were carried out with NAMD (65) using the all-atom CHARMM36 parameter set for the protein and sterol molecules (66) and the TIP3P model for water molecules (64). The solvated protein-membrane complex was simulated with molecular dynamics (MD) at 310 K according to the following protocol: 1) equilibration MD with Langevin dynamics (time step of 1fs) for 50ps followed by CPT dynamics (time step 2fs) for 350ps; 2) production MD with CPT dynamics (time step 2fs) for 100 ns. To simulate a continuous system, periodic boundary conditions were applied. Electrostatic interactions were summed with the Particle Mesh Ewald method (67) (grid spacing ~0.87Å; fftx 144, ffty 144, fftz 180). A nonbonded cutoff of 16.0 Å was used, and Heuristic testing was performed at each energy call to evaluate whether the non-bonded pair list should be updated.

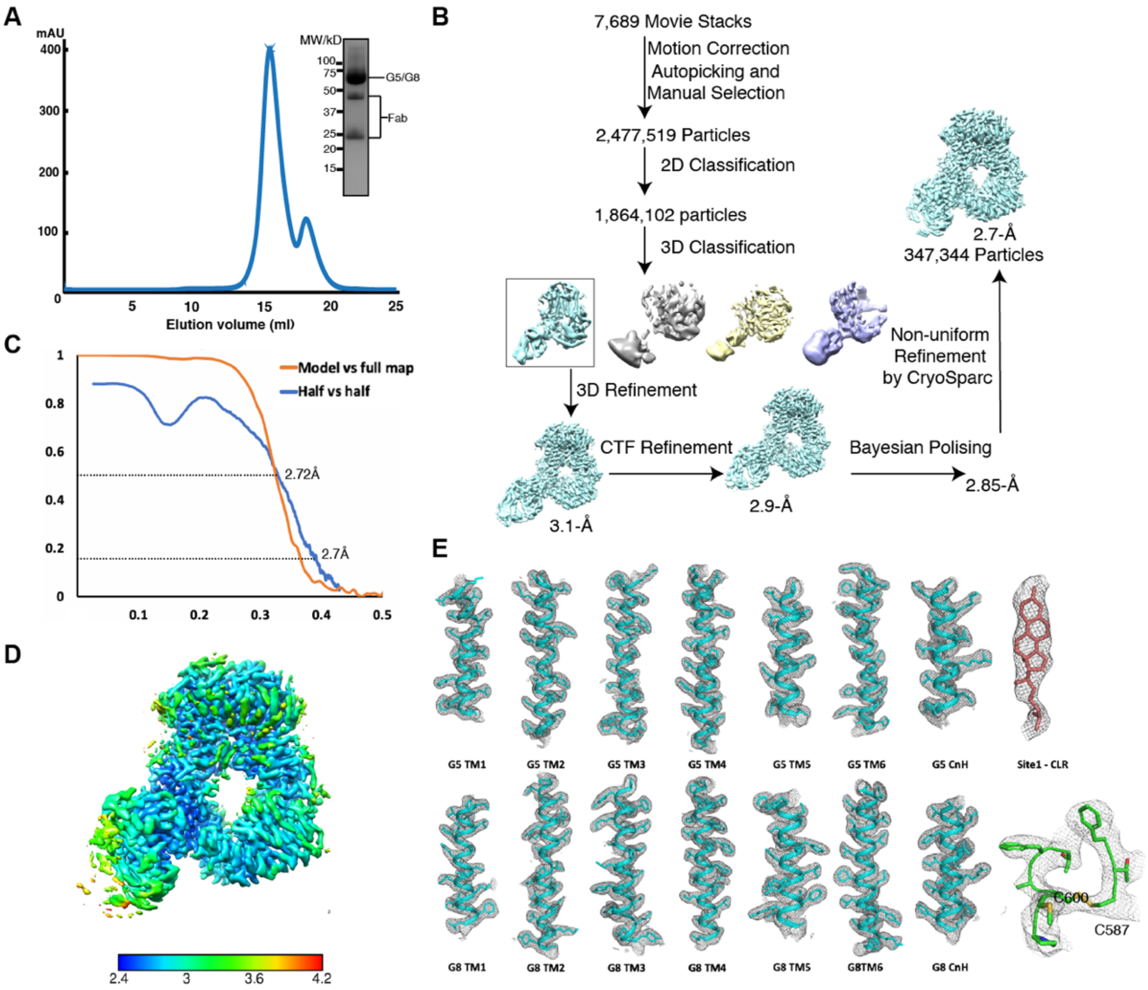

Fig. S1 Data processing and cryo-EM maps of G5G8 expressed in HEK cells.

(A) Representative Superose 6 increase 10/30 gel-filtration chromatogram of G5G8-Fab2C7 complex. The peak fraction is shown on SDS-PAGE with molecular markers. (B) The data processing workflow in RELION-3. The cryo-EM 3D classes as well as the masks used for refinement are shown. (C) Fourier shell correlation (FSC) curve as a function of resolution (orange) from RELION-3 and the FSC curve calculated between the refined structure model and the full map (blue) from Phenix. (D) Local resolution estimated using RELION-3. (E) The major structural element of G5G8. EM density maps are shown in mesh at 5σ level.

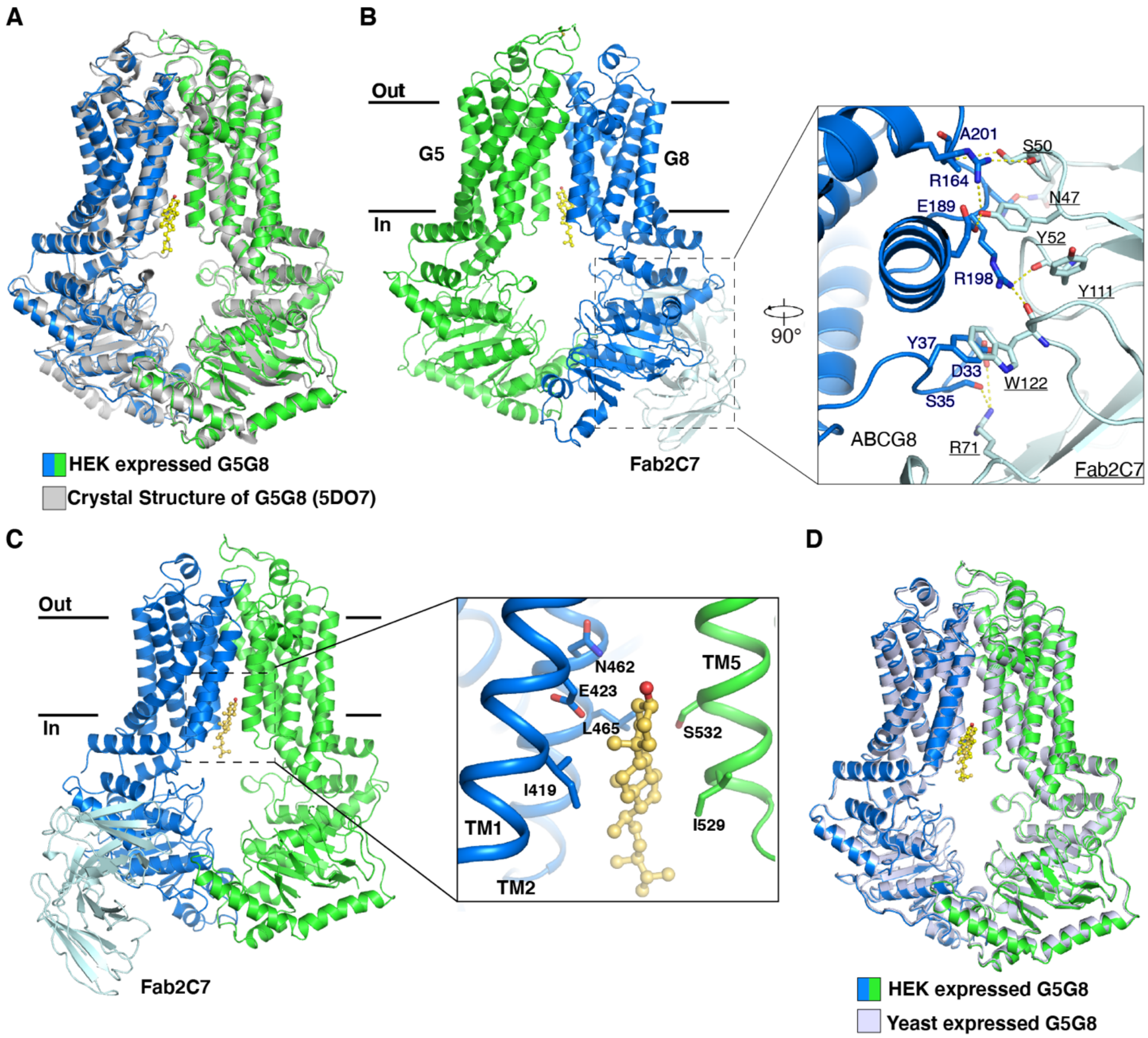

Fig. S2 Structural comparison of distinct states of G5G8.

(A) Structural comparison between HEK expressed G5G8 and previously determined crystal structure of G5G8 expressed from yeast. (B) The interaction details between Fab2C7 and G8. The residues that are involved in the interactions are shown in sticks. (C) Overall structure showing yeast expressed G5G8 bound to putative ergosterol viewed from the side of the membrane. The right panel shows interaction details of ergosterol (yellow sticks) in site 1 of G5G8. (D) Structural comparison between HEK expressed G5G8 and yeast expressed G5G8.

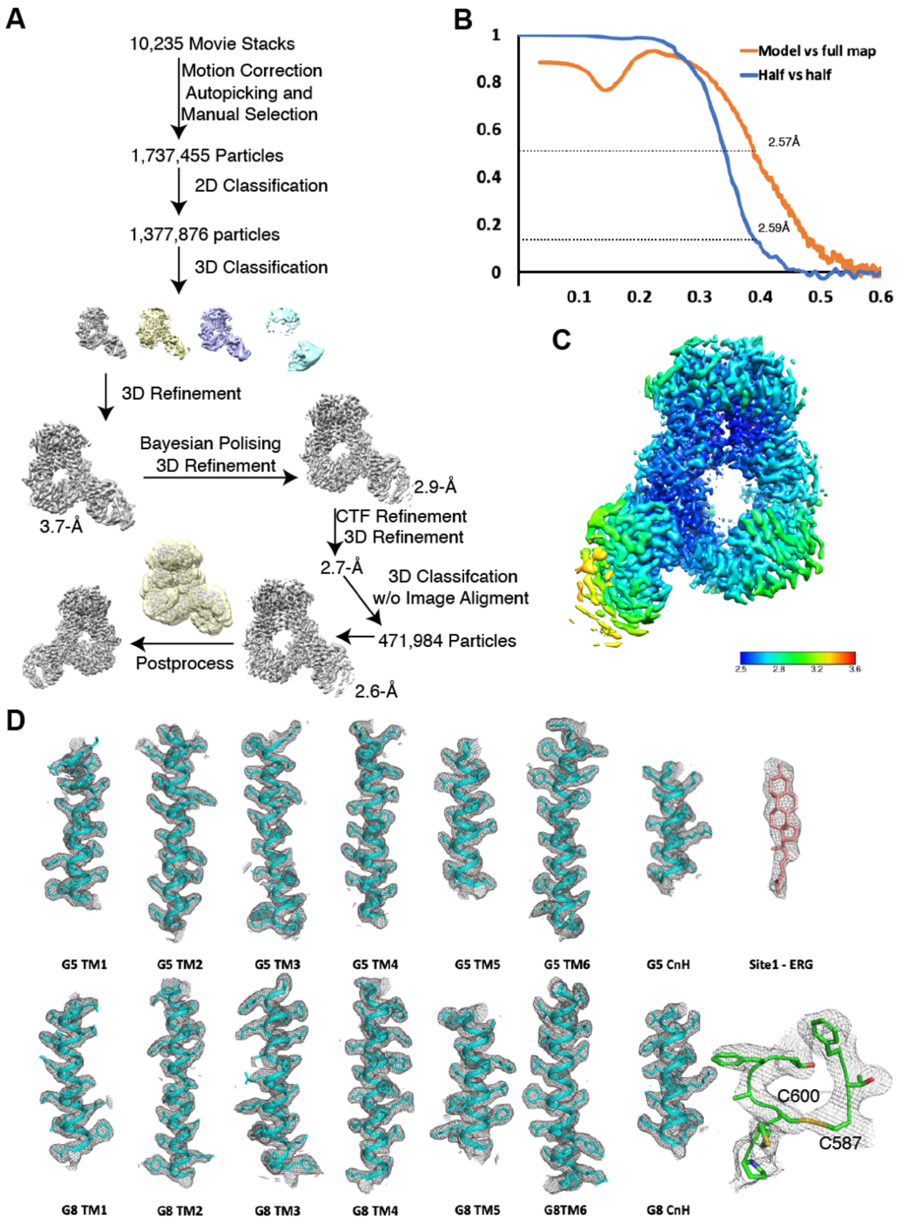

Fig. S3 Data processing and cryo-EM maps of yeast expressed G5G8.

(A) The data processing workflow in RELION-3. The cryo-EM 3D classes as well as the masks used for refinement are shown. (B) Fourier shell correlation (FSC) curve as a function of resolution (blue) from RELION-3 and the FSC curve calculated between the refined structure model and the full map (yellow) from Phenix. (C) Local resolution estimated using RELION-3. (D) The major structural element of G5G8. EM density maps are shown in mesh at 5σ level.

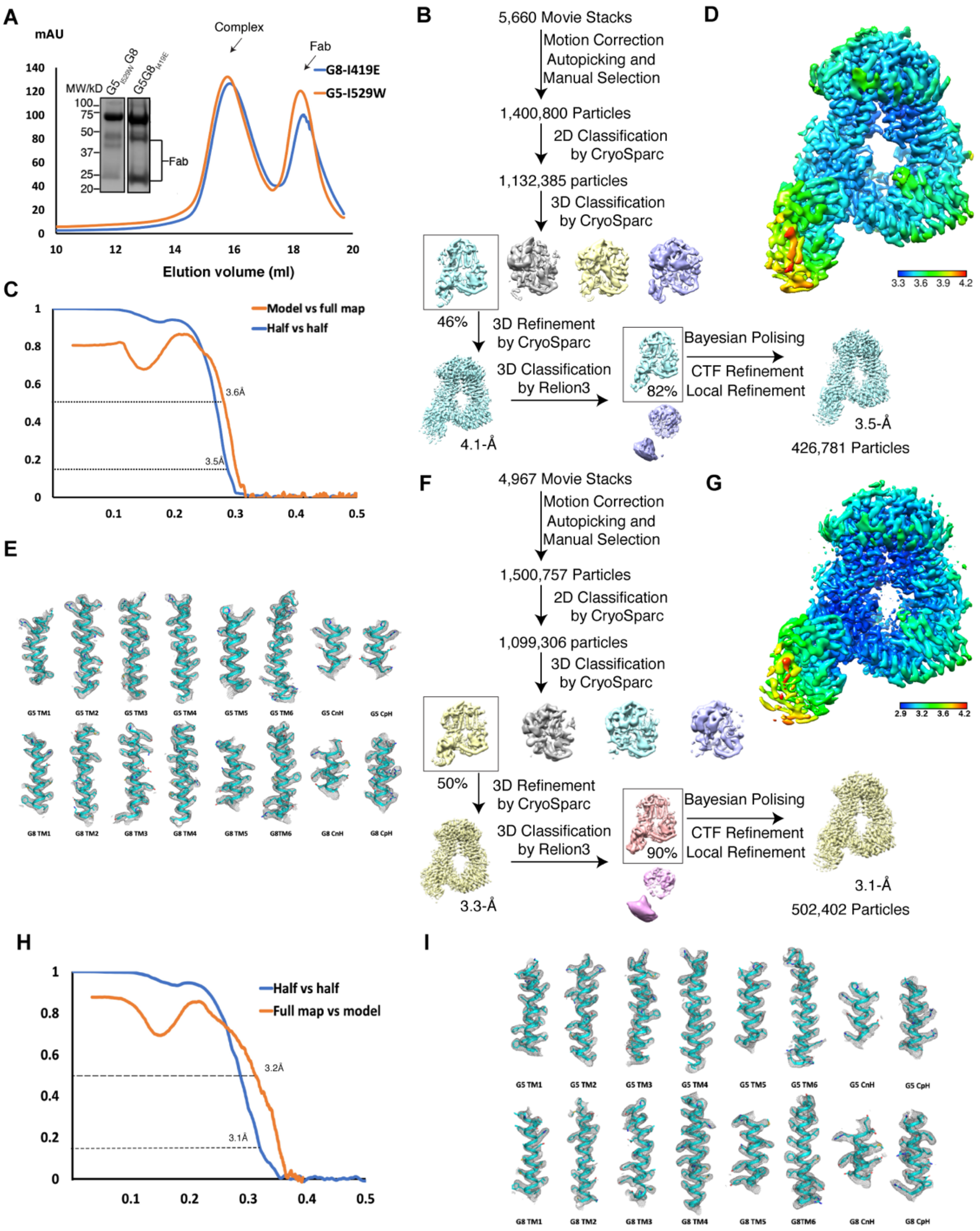

Fig. S4 Data processing and cryo-EM maps of G5$_{I529W}$G8 and G5G8$_{I419E}$ expressed in HEK cells.

(A) Representative Superose 6 increase 10/30 gel-filtration chromatogram of $G5_{I529W}G8$-Fab2C7 (orange) and $G5G8_{I419E}$-Fab2C7 (blue) complexes. The peak fraction is shown on SDS-PAGE with molecular markers. (B) and (F) The data processing workflow in RELION-3 of $G5_{I529W}G8$-Fab2C7 (panel B) and $G5G8_{I419E}$-Fab2C7 (panel F). The cryo-EM 3D classes as well as the masks used for refinement are shown. (C) and (H) Fourier shell correlation (FSC) curve as a function of resolution (blue) from RELION-3 and the FSC curve calculated between the refined structure model and the full map (yellow) from Phenix. The cures of $G5_{I529W}G8$-Fab2C7 are shown in panel C and the cures of $G5G8_{I419E}$-Fab2C7 are shown in panel H. (D) and (G) Local resolution estimated of $G5_{I529W}G8$-Fab2C7 (panel D) and $G5G8_{I419E}$-Fab2C7 (panel G) using RELION-3. (E) and (I) The major structural element of of $G5_{I529W}G8$-Fab2C7 (panel E) and $G5G8_{I419E}$-Fab2C7 (panel I). EM density maps are shown in mesh at 5σ level.

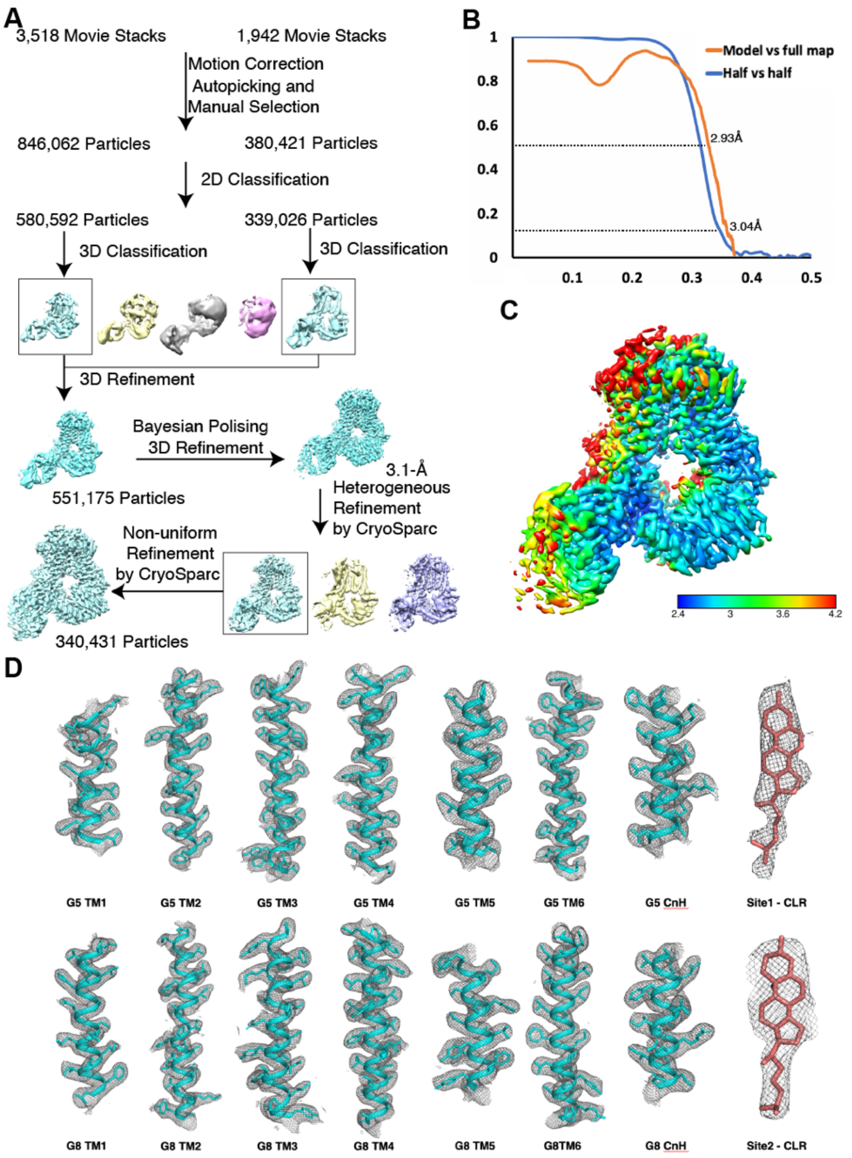

Fig. S5 Data processing and cryo-EM maps of cholesterol bound G5G8.

(A) The data processing workflow in RELION-3. The cryo-EM 3D classes as well as the masks used for refinement are shown. (B) Fourier shell correlation (FSC) curve as a function of resolution (blue) from RELION-3 and the FSC curve calculated between the refined structure model and the full map (yellow) from Phenix. (C) Local resolution estimated using RELION-3. (D) The major structural element of G5G8. EM density maps are shown in mesh at 5σ level.

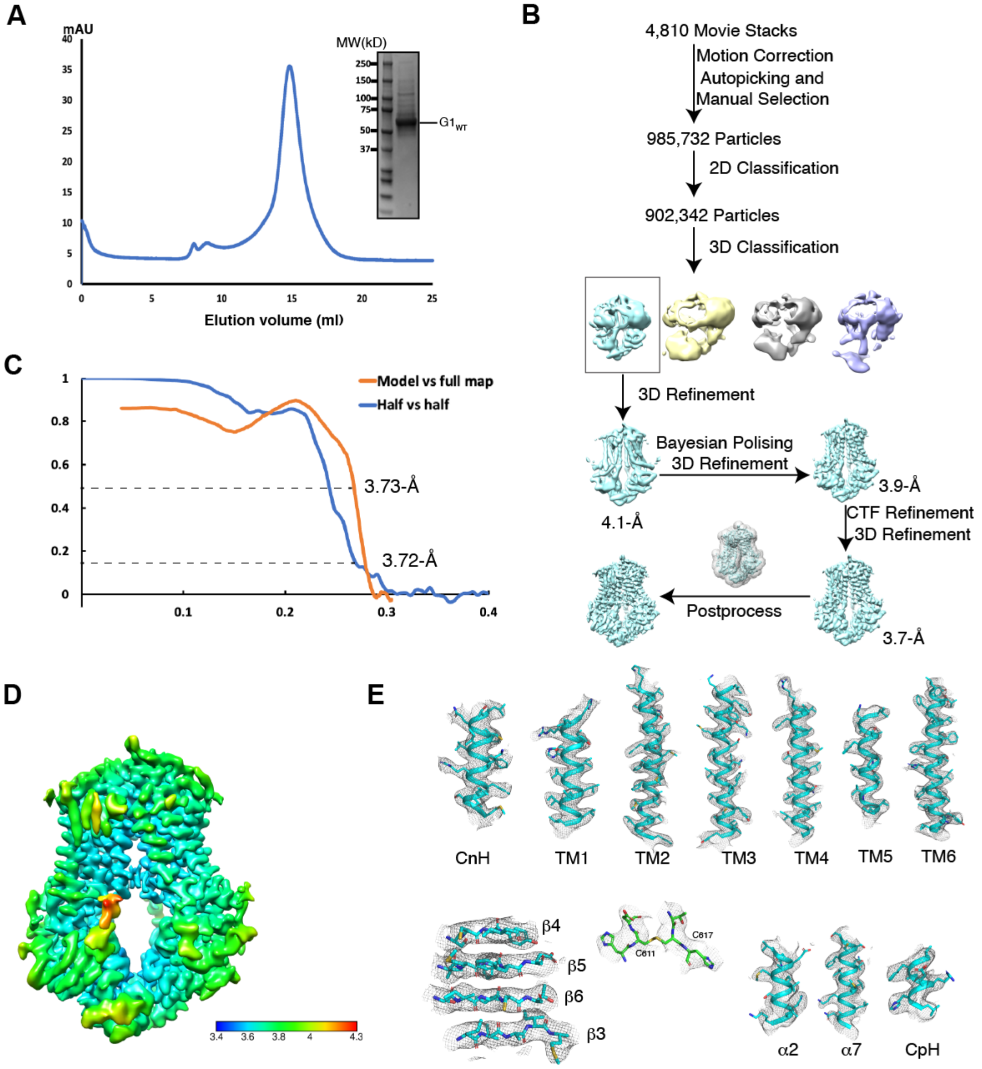

Fig. S6 Data processing and cryo-EM maps of G1WT.

(A) Representative Superose 6 increase 10/30 gel-filtration chromatogram of G1WT. The peak fraction is shown on SDS-PAGE with molecular markers. (B) The data processing workflow in RELION-3. The cryo-EM 3D classes as well as the masks used for refinement are shown. (C) Fourier shell correlation (FSC) curve as a function of resolution (blue) from RELION-3 and the FSC curve calculated between the refined structure model and the full map (orange) from Phenix. (D) Local resolution estimated using RELION-3. (E) The major structural element of G1WT. EM density maps are shown in mesh at 5σ level.

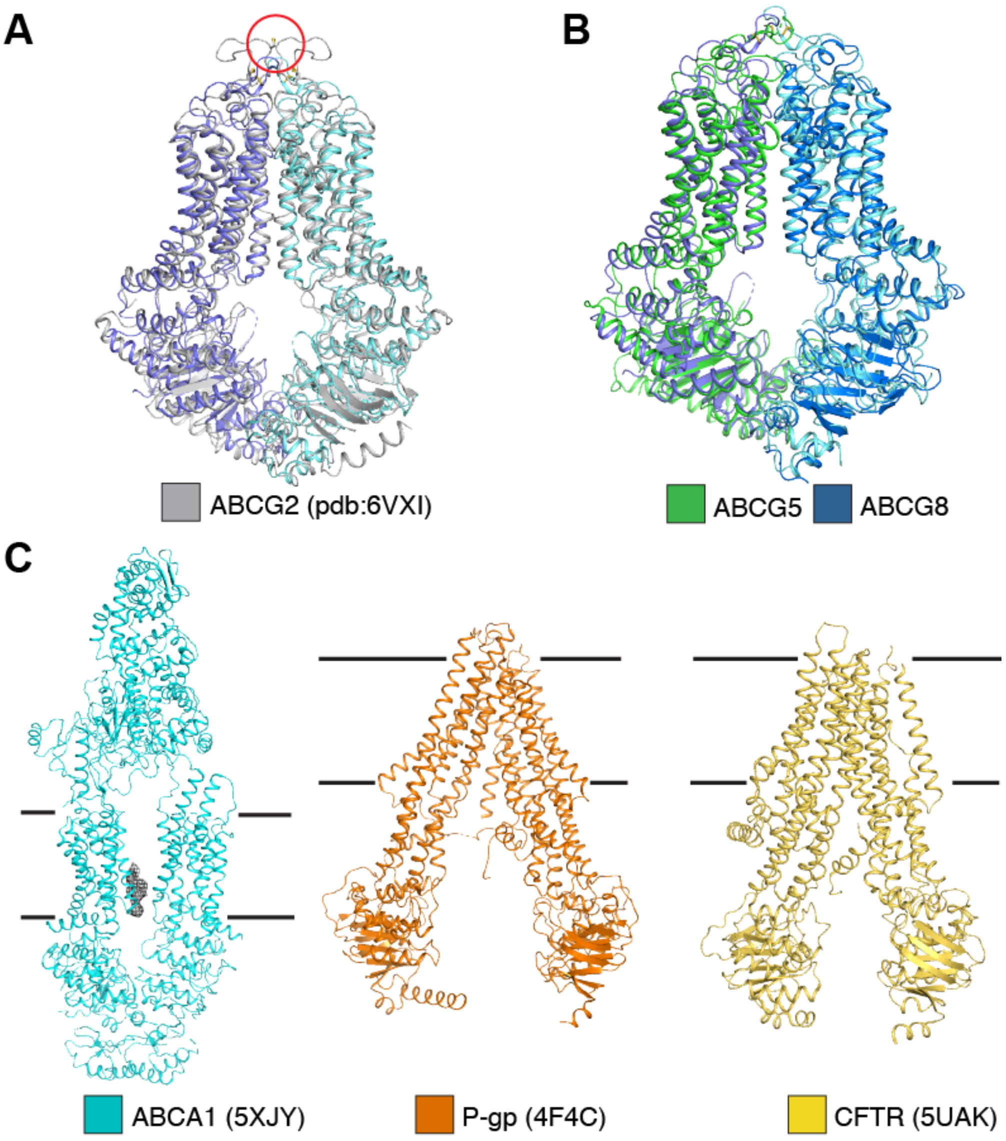

Fig. S7 Structural comparison of G1 with other ABC transporters.

(A) Structural comparison of G1 with ABCG2. The intermolecular disulfide bond of ABCG2 is indicated by a red circle. (B) Structural comparison of G1 with G5G8. (C) The structures of ABCA1 (left), P-glycoprotein (middle) and CFTR (right). The sterol-like density of ABCA1 is shown in gray mesh.

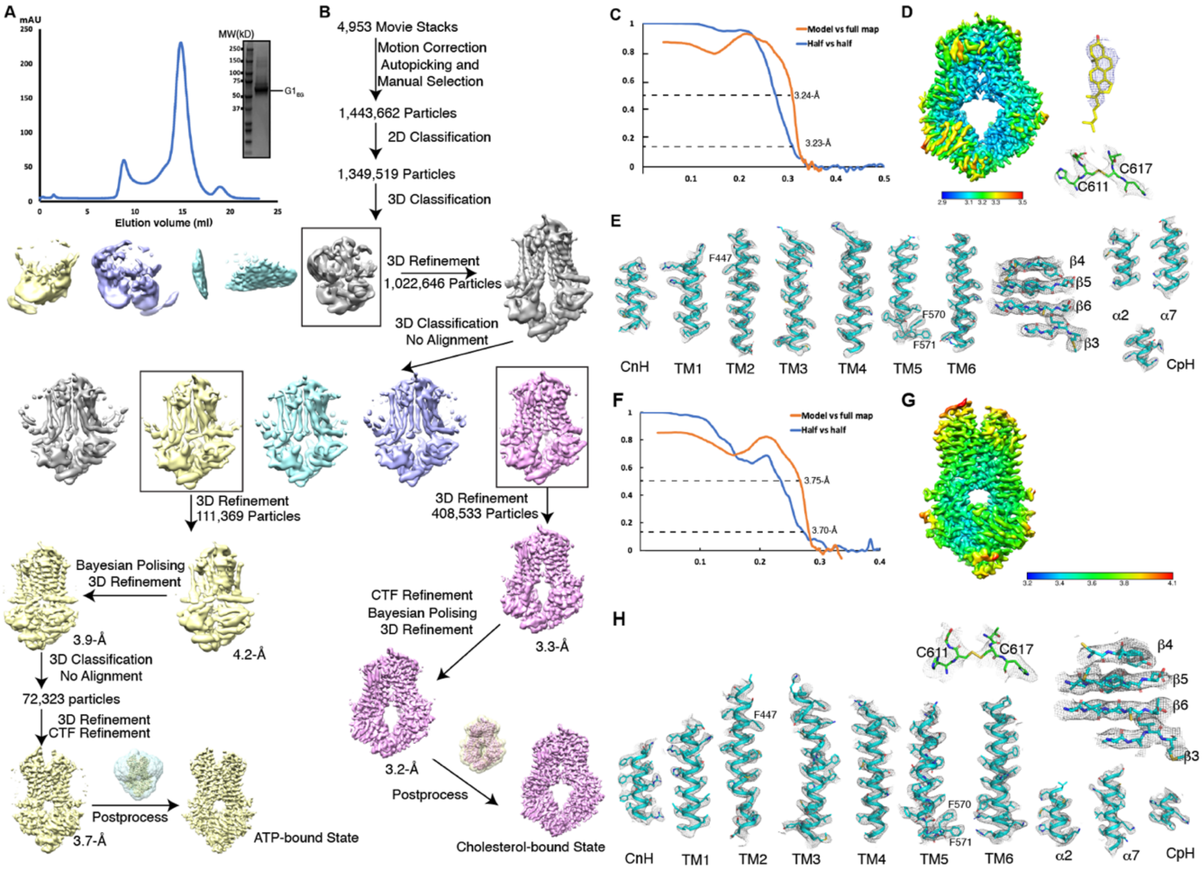

Fig. S8 Data processing and cryo-EM of G1$_{EQ}$.

(A) Representative Superose 6 increase 10/30 gel-filtration chromatogram of G1$_{EQ}$. The peak fraction is shown on SDS-PAGE with molecular markers. (B) The data processing workflow in RELION-3. The cryo-EM 3D classes as well as the masks used for refinement are shown. (C) and (F) Fourier shell correlation (FSC) curve as a function of resolution (blue) from RELION-3 and the FSC curve calculated between the refined structure model and the full map (orange) from Phenix. (D) and (G) Local resolution estimated using RELION-3. (E) and (H) The major structural element of G1$_{EQ}$. EM density maps are shown in mesh at 5σ level. The cholesterol-bound G1$_{EQ}$ in panel (C), (D) and (E); the ATP-bound GEQ in panel (F), (G) and (H).

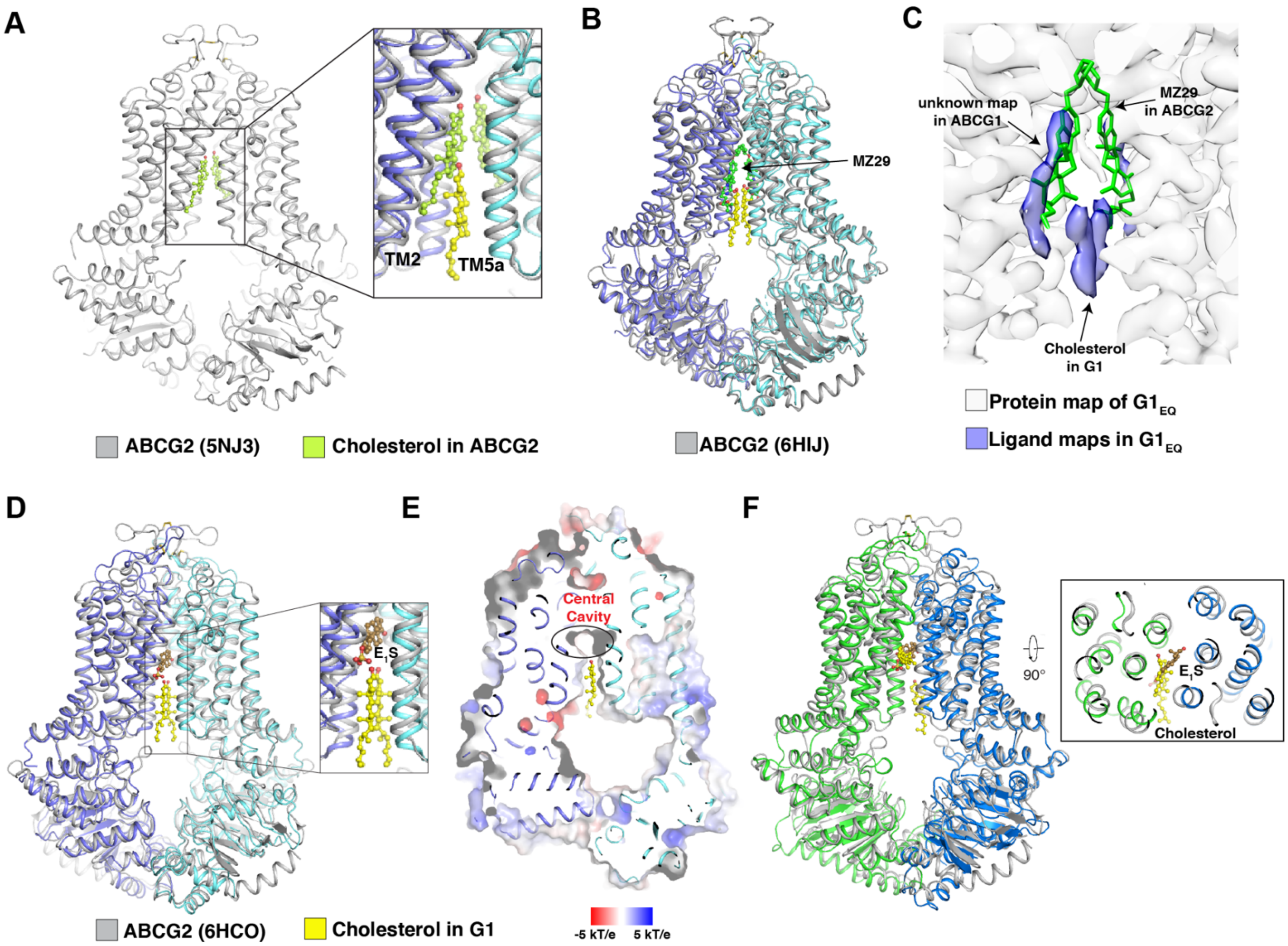

Fig. S9 Structural comparison of G1, G5G8 with ABCG2.

(A) The structure of ABCG2 bound to cholesterol. The putative cholesterol binding sites of G1 and ABCG2 is zoomed. (B) Comparison between cholesterol-bound G1 and MZ29-bound ABCG2. The ligands are shown in sticks. (C) The MZ29 ligand in ABCG2 (green sticks) with the cryo-EM map of cholesterol-bound G1EQ. The cryo-EM map is shown at 0.023 threshold level. (D) Comparison of cholesterol bound G1 and estrone 3-sulfate (E_1S) bound ABCG2. The binding site is zoomed in from the membrane view. (E) The electrostatic surface representation of the central cavity of G1 in the membrane. The central cavity is indicated by an oval. (F) Comparison of cholesterol-bound G5G8 and E_1S bound ABCG2. The binding site is zoomed in from the extracellular view.

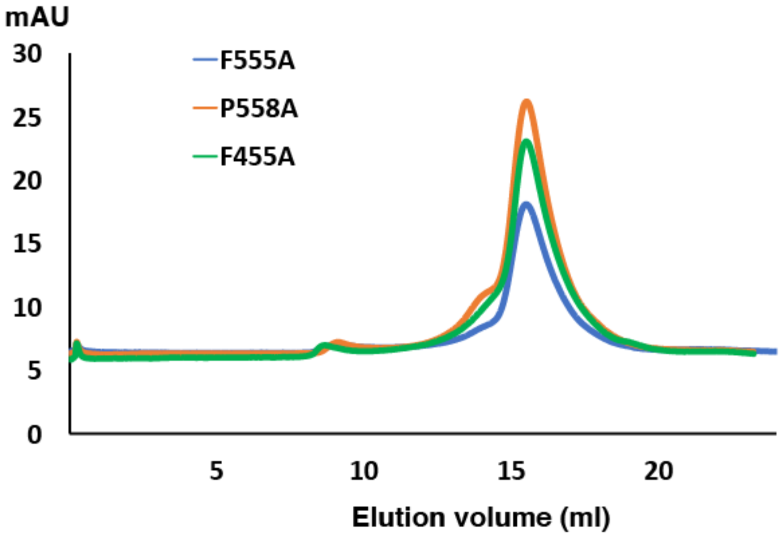

Fig. S10 Purification of human G1 variants for ATPase activity assays.

Representative Superose 6 increase 10/30 gel-filtration chromatograms of G1 variants in buffer containing 20 mM Hepes pH 7.5, 150 mM NaCl, and 0.06% (w/v) Digitonin.

Table S1 G5G8 Cryo-EM data collection, refinement and validation statistics.

	ABCG5/ABCG8<br>Yeast-apo<br>(EMDB-24312)<br>(PDB-7R89)	ABCG5/ABCG8<br>Hek293-apo<br>(EMDB-24313)<br>(PDB-7R8A)	ABCG5/ABCG8<br>Yeast-cholesterol<br>(EMDB-24314)<br>(PDB-7R8B)
**Data collection and processing**			
Magnification	105k	105k	105k
Voltage (kV)	300	300	300
Electron exposure (e–/Å^2)	60	60	60
Defocus range (μm)	1.0 to 2.0	0.8-2.0	1.0 to 2.0
Pixel size (Å)	0.832	0.844	0.833
Symmetry imposed	C1	C1	C1
Initial particle images (no.)	1,737,455	2,477,519	1,443,662
Final particle images (no.)	471,984	187,255	346,321
Map resolution (Å)	2.6	2.7	3.0
FSC threshold	0.143	0.143	0.143
**Refinement**			
Model resolution (Å)	2.6	3.0	3.0
FSC threshold	0.5	0.5	0.5
Map sharpening *B* factor (Å^2)	-30	-50	-50
Model composition			
Non-hydrogen atoms	10530	10527	10868
Protein residues	1332	1332	1371
Ligands	1	1	2
B factors (Å^2)			
Protein	108.85	84.68	81.44
Ligand	72.42	82.01	74.97
R.m.s. deviations			
Bond lengths (Å)	0.002	0.002	0.002
Bond angles (°)	0.520	0.552	0.502
Validation			
MolProbity score	2.05	2.07	1.81
Clashscore	7.12	6.23	6.57
Poor rotamers (%)	3.39	3.39	1.94
Ramachandran plot			
Favored (%)	96.18	95.34	96.52
Allowed (%)	3.82	4.66	3.48
Disallowed (%)	0.0	0.00	0.0

Table S2 G5G8 mutants Cryo-EM data collection, refinement and validation statistics.

	ABCG5/ABCG8$_{I419E}$ Hek293-apo (EMDB-24310) (PDB-7R87)	ABCG5$_{I529W}$/ABCG8 Hek293-apo (EMDB-24311) (PDB-7R88)
**Data collection and processing**		
Magnification	105k	105k
Voltage (kV)	300	300
Electron exposure (e–/Å^2)	60	60
Defocus range (μm)	1.0 to 2.0	1.0 to 2.0
Pixel size (Å)	0.833	0.833
Symmetry imposed	C1	C1
Initial particle images (no.)	1,500,757	1,400,800
Final particle images (no.)	502,402	426,781
Map resolution (Å)	3.1	3.5
FSC threshold	0.143	0.143
**Refinement**		
Model resolution (Å)	3.2	3.6
FSC threshold	0.5	0.5
Map sharpening *B* factor (Å^2)	-80	-80
Model composition		
Non-hydrogen atoms	10799	10787
Protein residues	1370	1368
Ligands	0	0
B factors (Å^2)		
Protein	74.85	66.26
Ligand	N/A	N/A
R.m.s. deviations		
Bond lengths (Å)	0.001	0.002
Bond angles (°)	0.400	0.433
Validation		
MolProbity score	1.48	1.51
Clashscore	5.43	5.25
Poor rotamers (%)	0.76	1.02
Ramachandran plot		
Favored (%)	96.89	96.51
Allowed (%)	3.11	3.49
Disallowed (%)	0.0	0.0

Table S3 G1 Cryo-EM data collection, refinement and validation statistics.

	$G1_{WT}$-*apo* (EMDB-24315) (PDB-7R8C)	$G1_{EQ}$-cholesterol (EMDB-24316) (PDB-7R8D)	$G1_{EQ}$-ATP (EMDB-24317) (PDB-7R8E)
**Data collection and processing**			
Magnification	105k	105k	105k
Voltage (kV)	300	300	300
Electron exposure (e–/Å^2)	60	60	60
Defocus range (μm)	1.2 to 2.5	1.0 to 2.0	1.0 to 2.0
Pixel size (Å)	0.833	0.833	0.833
Symmetry imposed	C2	C2	C2
Initial particle images (no.)	987,532	1,443,662	1,443,662
Final particle images (no.)	545,471	408,553	72,323
Map resolution (Å)	3.7	3.2	3.7
FSC threshold	0.143	0.143	0.143
**Refinement**			
Model resolution (Å)	3.7	3.2	3.8
FSC threshold	0.5	0.5	0.5
Map sharpening *B* factor (Å^2)	-100	-151.3	-130.9
Model composition			
Non-hydrogen atoms	8540	8764	8702
Protein residues	1090	1090	1096
Ligands	0	8	6
B factors (Å^2)			
Protein	109.89	57.26	133.48
Ligand	N/A	57.79	92.71
R.m.s. deviations			
Bond lengths (Å)	0.002	0.002	0.015
Bond angles (°)	0.478	0.497	1.831
Validation			
MolProbity score	1.69	1.88	2.18
Clashscore	6.38	6.34	8.66
Poor rotamers (%)	0.21	1.93	1.71
Ramachandran plot			
Favored (%)	95.18	95.55	90.41
Allowed (%)	4.82	4.45	9.59
Disallowed (%)	0.0	0.0	0.0

Movie S1 Molecular dynamics simulations of sterol from site 2 of G5G8 to extracellular cavity in 100 ns time scale.

The sterol subtract is shown in stick. The G5 and G8 are colored in green and blue, respectively.

SI References

49. X. Li *et al.*, Electron counting and beam-induced motion correction enable near-atomic-resolution single-particle cryo-EM. *Nature methods* **10**, 584-590 (2013).
50. A. Rohou, N. Grigorieff, CTFFIND4: Fast and accurate defocus estimation from electron micrographs. *Journal of structural biology* **192**, 216-221 (2015).
51. J. Zivanov *et al.*, New tools for automated high-resolution cryo-EM structure determination in RELION-3. *eLife* **7** (2018).
52. A. Punjani, J. L. Rubinstein, D. J. Fleet, M. A. Brubaker, cryoSPARC: algorithms for rapid unsupervised cryo-EM structure determination. *Nature methods* **14**, 290-296 (2017).
53. A. Waterhouse *et al.*, SWISS-MODEL: homology modelling of protein structures and complexes. *Nucleic acids research* **46**, W296-W303 (2018).
54. N. M. I. Taylor *et al.*, Structure of the human multidrug transporter ABCG2. *Nature* **546**, 504-509 (2017).
55. P. Emsley, K. Cowtan, Coot: model-building tools for molecular graphics. *Acta crystallographica. Section D, Biological crystallography* **60**, 2126-2132 (2004).
56. P. D. Adams *et al.*, PHENIX: a comprehensive Python-based system for macromolecular structure solution. *Acta crystallographica. Section D, Biological crystallography* **66**, 213-221 (2010).
57. G. N. Murshudov, A. A. Vagin, E. J. Dodson, Refinement of macromolecular structures by the maximum-likelihood method. *Acta crystallographica. Section D, Biological crystallography* **53**, 240-255 (1997).
58. V. B. Chen *et al.*, MolProbity: all-atom structure validation for macromolecular crystallography. *Acta crystallographica. Section D, Biological crystallography* **66**, 12-21 (2010).
59. E. F. Pettersen *et al.*, UCSF Chimera--a visualization system for exploratory research and analysis. *Journal of computational chemistry* **25**, 1605-1612 (2004).
60. B. R. Brooks *et al.*, CHARMM: A program for macromolecular energy, minimization, and dynamics calculations. *Journal of computational chemistry* **4**, 30 (1983).
61. G. Kieseritzky, E. W. Knapp, Optimizing pKa computation in proteins with pH adapted conformations. *Proteins* **71**, 1335-1348 (2008).
62. S. Jo, T. Kim, V. G. Iyer, W. Im, CHARMM-GUI: a web-based graphical user interface for CHARMM. *Journal of computational chemistry* **29**, 1859-1865 (2008).
63. M. A. Lomize, A. L. Lomize, I. D. Pogozheva, H. I. Mosberg, OPM: orientations of proteins in membranes database. *Bioinformatics* **22**, 623-625 (2006).
64. W. Jorgensen, J. Chandrasekhar, J. Madura, R. Impey, M. Klein, Comparison of simple potential functions for simulating liquid water. *The Journal of Chemical Physics* **79**, 9 (1983).
65. J. C. Phillips *et al.*, Scalable molecular dynamics on CPU and GPU architectures with NAMD. *J Chem Phys* **153**, 044130 (2020).
66. A. D. MacKerell *et al.*, All-atom empirical potential for molecular modeling and dynamics studies of proteins. *J Phys Chem B* **102**, 3586-3616 (1998).
67. U. Essmann *et al.*, A smooth particle mesh Ewald method. *J. Chem. Phys.* **103**, 16 (1995).
